# Novel Dorsomorphin Derivatives: Molecular Modeling, Synthesis, and Bioactivity Evaluation

**DOI:** 10.3390/biom16010145

**Published:** 2026-01-14

**Authors:** Evangelia N. Tzanetou, Sandra Liekens, Konstantinos M. Kasiotis, Nikolas Fokialakis, Nikolaos Tsafantakis, Raul SanMartin, Haralampos Tzoupis, Konstantinos D. Papavasileiou, Antreas Afantitis, Serkos A. Haroutounian

**Affiliations:** 1 Laboratory of Nutritional Physiology and Feeding, Department of Animal Sciences and Aquaculture, Agricultural University of Athens, Iera Odos 75, 11855 Athens, Greece; ev.tzanetou@bpi.gr (E.N.T.); k.kasiotis@bpi.gr (K.M.K.); 2 Rega Institute for Medical Research, KU Leuven, Minderbroedersstraat 10, B-3000 Leuven, Belgium; sandra.liekens@kuleuven.be; 3 Department of Pharmacognosy and Natural Products Chemistry, Faculty of Pharmacy, University of Athens, Panepistimiopolis, Zografou, 15771 Athens, Greece; fokialakis@pharm.uoa.gr (N.F.); ntsafantakis@pharm.uoa.gr (N.T.); 4 Department of Organic and Inorganic Chemistry, Faculty of Science and Technology, University of the Basque Country, (UPV-EHU), 48940 Leioa, Spain; raul.sanmartin@ehu.eus; 5 Department of ChemoInformatics, NovaMechanics Ltd., Nicosia 1070, Cyprus; tzoupis@novamechanics.com (H.T.); papavasileiou@novamechanics.com (K.D.P.); afantitis@novamechanics.com (A.A.); 6 Department of ChemoInformatics, NovaMechanics MIKE, 18545 Piraeus, Greece; 7 Division of Data Driven Innovation, Entelos Institute, Larnaca 6059, Cyprus; 8 Department of Pharmacy, Frederick University, Nicosia 1036, Cyprus

**Keywords:** dorsomorphin, pyrazolo[1,5-*a*]pyrimidine, microwave-assisted synthesis, antiproliferative, molecular modeling

## Abstract

Dorsomorphin, a pyrazolo[1,5-*a*]pyrimidine derivative, inhibits the bone morphogenetic protein (BMP) pathway by targeting the type I BMP receptors active in receptor-like kinases. However, the investigation of its—and its derivatives’—antiproliferative activity towards endothelial and cancer cell lines still requires reinforcement with additional studies. In the presented work, several dorsomorphin derivatives have been efficiently synthesized, based on a previously reported synthetic protocol with minor modifications. The endeavor was reinforced by a molecular docking study on the interactions of the designed derivatives with various protein targets, while the inhibitory effects of the synthesized novel molecules on the proliferation of murine leukemia cells (L1210), human T-lymphocyte cells (CEM), human cervix carcinoma cells (HeLa), and endothelial cells (human dermal microvascular, HMEC-1, and bovine aortic endothelial cells, BAECs) were investigated. Among the compounds tested, diphenol **22**, emerged as the most promising bioactive lead since it demonstrated half-maximal inhibitory concentration (IC_50_) values below 9 μM in all tested lines except HeLa cells. In the same context, the carbamate derivative **6** was determined as a potent inhibitor of endothelial cell proliferation in BAECs at a low micromolar range. In conclusion, the presented work not only reveals promising antiproliferative dorsomorphin derivatives but also sets the basis for further exploitation of dorsomorphin’s bioactive portfolio, based on bioactivity results and molecular modeling calculations.

## 1. Introduction

The pyrimidine moiety constitutes a building block of both DNA and RNA. Pyrimidine derivatives are widely known to demonstrate a broad range of diverse pharmacological activities, including anticancer [1], antiviral (especially anti-HIV) [2], antimicrobial, and anti-inflammatory properties [3]. In addition, they also display pronounced activity against gonadotropin-releasing hormone receptors, as well as herbicidal activity targeting aceto-hydroxy acid synthase, which catalyzes the initiating event in branched-chain amino acid biosynthesis [4].

The significant number of patents published during the last decade (i.e., 59) for various pyrimidine-based anticancer agents is indicative that the specific heterocyclic ring constitutes an emerging research subject on a global scale. It must be noted that 32 of these patents have appeared during the last decade, highlighting the interest of the research community for the development of pyrimidine-based anticancer agents because of the promising activities displayed by these scaffolds as potential future drug candidates [1].

**Figure 1:**
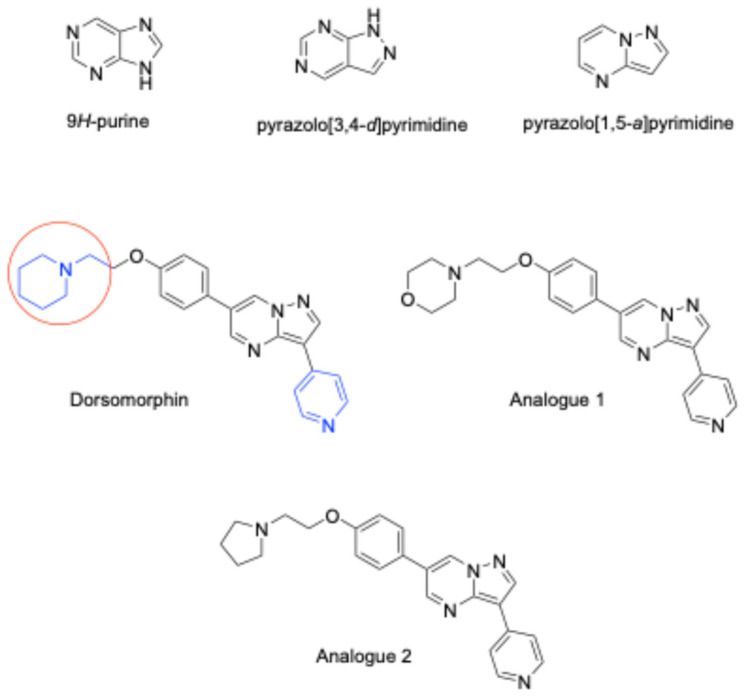
Structural backbone of 9H-purines, pyrazolopyrimidines, dorsomorphin, and analogs (structural modifications are highlighted with red and blue).

Among the various novel pyrimidine derivatives developed and tested to date, pyrazolo[3,4-*d*]pyrimidines are considered biologically active isomeric analogs of purines (purine structure shown in Figure 1), since their structural backbone closely resembles the purine scaffold, with a pyrazole ring instead of the imidazole moiety found in purines (Figure 1). Previous endeavors have consistently established the anticancer and/or antiviral bioactivities of many purine nucleosides and nucleotides, which are currently marketed as valacyclovir, acyclovir, famciclovir, and cladribine inter alia [5,6]. In this respect, the 1*H*-pyrazolo[3,4-*d*]pyrimidines isomers of purines are widely used as purine replacements in the synthesis of bioactive molecules, aiming to improve their targeted activities [7]. In this context, several pyrazolo[3,4-*d*]pyrimidine derivatives have been found to display potent anticancer and antileukemic activities [8,9], acting via different mechanisms such as cyclin-dependent kinase inhibitors, tyrosine kinase inhibitors, potent xanthine oxidase inhibitors, and/or adenosine receptor antagonists.

Pyrazolo[1,5-*a*]pyrimidines comprise a category of compounds encompassing a similar condensed bi-heterocyclic backbone, which has also been widely exploited from both chemical and pharmacological perspectives. The literature contains numerous papers concerning the inhibitory activities of diverse pyrazolo[1,5-*a*]pyrimidine derivatives on phosphodiesterases [1,10], along with their anti-inflammatory properties and anxiolytic activities, which do not enhance the depressant effects of ethanol or barbiturates on the central nervous system [11].

Dorsomorphin dihydrochloride (DOS, Figure 1) is a pyrazolo[1,5-*a*]pyrimidine molecule that is able to disrupt dorsoventral axis formation in zebrafish and acts as a potent selective inhibitor of AMP-activated protein kinase (AMPK) and bone morphogenic protein (BMP) signaling [12]. The latter plays a divergent and critical role in embryonic pattern construction and is implicated in the development of several diseases. In addition, BMP signaling also coordinates developmental patterning, playing essential physiological roles in mature organisms [13]. As a small molecule, DOS has been shown to stimulate mouse embryonic fibroblasts to differentiate into neural crest-like precursors [14], while several DOS analogs have recently been synthesized and reported to selectively inhibit the Janus family of cytoplasmic tyrosine kinase domains [15]. To this end, the medicinal attributes of DOS derivatives with respect to their anticancer activities have been reviewed by Ismail and coworkers [16]. In parallel, there is ongoing interest in the synthesis and bioactivity evaluation of novel DOS analogs and derivatives, such as DOS analogs 1 and 2 depicted in Figure 1, adapted from Novikova et al., 2025 [17].

Encouraged by the aforementioned findings and as a part of our ongoing interest to develop novel structural leads with potent chemotherapeutic activities, we aimed to design, synthesize, and exploit the anticancer activities of various novel pyrazolopyrimidines. As a starting point of our design, we utilized previous reports revealing the antineoplastic activities of substituted hydrazines [18] to design molecules containing hydrazine and amino functionalities. The incorporation of an amide moiety was inspired by the fact that many antitumor antibiotics, such as bleomycin [19] and pyrazofurin, incorporate this moiety into their structural scaffolds, while the chemotherapeutic activities associated with many hydrazones motivated the synthesis of novel hydrazines, which were further cyclized into novel tricyclic compounds, aiming to exploit their antitumor and antimicrobial activities. Additionally, since various triazoles are known to possess potent antineoplastic activities [20], we incorporated the triazole ring into a series of pyrazolopyrimidines, aiming to improve their bioactivity. Finally, the diverse chemotherapeutic activities [21,22] associated with various imidazole and pyrimidine derivatives inspired us to establish tricyclic ring derivatives that incorporate the imidazole or pyrimidine ring system fused to the core pyrazolopyrimidine nucleus, aiming to elucidate the activity of such compounds.

In this study, we designed and synthesized various novel analogs of DOS using the 4-phenyl-1*H*-pyrazol-5-amine as the key intermediate. This served as the core structure for the construction of novel DOS derivatives, with modifications of the DOS structure at applied the piperidin-1-yl and pyridin-4-yl moieties (see Figure 1, colored insertions). Considering the importance of molecular modeling in pharmaceutical and biomedical research [23] for elucidating the mechanisms of bioactive molecules, the newly synthesized DOS derivatives were evaluated at the atomistic level with respect to their interaction with various protein targets. For this purpose, we conducted a series of molecular docking calculations, focusing on enzymes implicated in BMP pathways and the inhibition of the Dickkopf-1 (DKK-1) protein in breast cancer cell lines. In addition, we also explored the interactions between novel compounds and the ABC type transporter, which plays a crucial role in transporting various substances out of cells through ATP-dependent mechanisms. Consequently, the antiproliferative activities of the synthesized intermediates and end products were determined, using a panel of endothelial and cancer cell lines and considering the described “off-target” effects to assess the effect of aromatic ring substitution on their respective bioactivities.

## 2. Materials and Methods

### 2.1. General Experimental Conditions

All reactions were performed in oven-dried glassware under argon atmosphere. All commercially available reagents and solvents were purchased from Aldrich Chemical Co. (St. Luis, MO, USA) or Fisher Scientific Co. (Waltham, MA, USA). All solvents were purified by distillation prior to use, while solvent mixtures used in chromatography were prepared at volume-to-volume ratios. Thin-layer chromatography (TLC) was performed on Merck precoated aluminum plates coated with silica gel 60 F_254_. Products’ visualization was realized by UV absorbance at 254 nm and by spraying an alcoholic solution of anisaldehyde and heating. Flash column chromatography was performed on SDS silica gel (35–70 μm). Melting points (Mps) were determined on a Stuart apparatus (SMP3, Keison Products, Chelmsford, Essex, UK) and are uncorrected. IR spectra were recorded on a Thermo electron corporation Nicolet 6700 FT-IR spectrometer (Waltham, MA, USA) using CH_2_Cl_2_ in ZnSe round windows. The reaction products were identified by recording their ^1^H NMR and ^13^C NMR spectra, at 400 and 50 MHz respectively, on Bruker DRX-400 and DRX-200 spectrometers (Billerica, MA, USA) in the indicated solvents (for NMR, see Figures S1–S46). Chemical shifts (δ) for proton and carbon resonances are quoted in parts per million (ppm) relative to tetramethylsilane (TMS) and deuterated chloroform (CDCl_3_), respectively, which were used as internal standards. The synthetic procedures and NMR data presented herein are reproduced, for convenience, from the respective PhD thesis conducted at the Agricultural University of Athens, Greece [24].

### 2.2. Indicative Procedure for the Synthesis of Pyrazolo[1,5-a]pyrimidines

*3,6-Bis(4-methoxyphenyl)pyrazolo[1,5-a]pyrimidine* (**4**): 4-Methoxyphenylacetonitrile (2 mL, 14.7 mmol) was dissolved in a mixture of 36 mL of DMFDMA/DMF/PhCF_3_ (1:1:2) in a microwave vessel, which was placed in a microwave-assisted synthesizer and heated at 400 Watts until TLC (n-hexane/EtOAc, 7:3) confirmed that the starting material was consumed (approximately 20 min). Afterward, a solution of hydrazine (7.3 mL, 150 mmol) diluted in 100 mL of a 0.9:0.1:1 mixture of EtOH/H_2_O/CH_3_COOH was added via syringe, and the resulting mixture was heated in a microwave for 10 min at 800 Watts. The reaction progress was supervised with TLC, and after its completion, the mixture was extracted twice with EtOAc. The combined organic layers were washed with brine, dried over Na_2_SO_4_, and the solvent was evaporated under reduced pressure. The resultant residue was purified by flash column chromatography using CH_2_Cl_2_ as the eluent, yielding 2020 mg of compound **4** (10.67 mmol, 81% yield) as pale yellow crystalline needles (Mp: 162 °C). The spectroscopic data were consistent with those reported in the literature.

*3,6-Bis(4-methoxyphenyl)pyrazolo[1,5-a]pyrimidine* (**21**): A solution of 150 mg (0.79 mmol) of 4-(4-methoxyphenyl)-1H-pyrazol-5-amine **20**, obtained directly without purification through the reaction of 4-methoxyphenylacetonitrile with hydrazine in DMFDMA, was dissolved in 7 mL of 10% AcOH/EtOH. To this solution, 140.8 mg (0.79 mmol) of 2-(4-methoxyphenyl)malondialdehyde was added. The mixture was placed in a microwave bath and irradiated for 10 min at 400 Watts. When the completion of the reaction was verified with TLC, the mixture was partitioned between EtOAc and H_2_O. The water layer was re-extracted with EtOAc, and the joint organic fractions were washed with brine, dried over Na_2_SO_4_, and evaporated under reduced pressure. To purify the resulting residue, flash column chromatography was applied using CH_2_Cl_2_ as the eluent, yielding 240 mg of compound **21** (0.72 mmol, yield: 97%) as yellow crystalline needles (Mp: 172 °C). **^1^H NMR** (CDCl_3_): 8.82 (2H, s, H-6, H-4), 8.46 (1H, s, H-1), 7.98 (2H, d, J 7.8 Hz, H-1′, H-5′), 7.60 (2H, d, J 8.4 Hz, H-1″, H-5″), 7.09 (2H, d, J 7.5 Hz, H-2″, H-4″), 7.05 (2H, d, J 7.5 Hz, H-2′, H-4′), H-2″, H-4″), 3.81 (3H, s, OCH_3_), 3.80 (3H, s, OCH_3_). **^13^C NMR** (Acetone-d6): δ = 149.0 (C-4), 142.17 (C-1), 131.0 (C-6), 110.74 (C-2), 127.65 (C-1′, C-5′), 128.08 (C-1″ και C-5″), 115.48 (C-2″, C-4″), 114.41 (C-2′, C-4′), 124.4 (C-6′), 126.06 (C-6″), 158. 92 (C-3′), 160.16 (C-3″), 122.43 (C-3), 55.63 (OCH_3_).

*3-Phenyl-6-(pyrazin-2-yl)pyrazolo[1,5-a]pyrimidine* (**14**): Compound **3** (150 mg, 0.94 mmol) was dissolved in 7 mL of 10% AcOH/EtOH, and 2-(4-pyrimidyl)malondialdehyde (141.2 mg, 0.94 mmol) was added. The mixture was reacted according to the general procedure for the synthesis of pyrazolopyrimidine derivatives. Pyrimidine **14** was obtained as pale yellow crystals (200 mg, 0.73 mmol, yield: 78%) (Mp: 117 °C). **^1^H NMR** (CDCl_3_): 9.33 (1H, d, J 2.4 Hz, H-6), 9.14 (1H, d, J 2.4 Hz H-4), 9.04 (1H, s, H-3″), 8.63 (1H, s, H-1″), 8.55 (1H, s, H-2″), 8.47 (1H, s, H-1), 8.0 (2H, d, J 8.4 Hz, H-1′, H-5′), 7.42 (3H, t, J 7.8 Hz, H-2′, H-3′, H-4′). **^13^C NMR** (Acetone-d6): *δ* = 134.08 (C-6), 144.82 (C-1), 147.71 (C-4), 141.3 (C-3″), 144.82 (C-1″), 144.82 (C-2″). 111.68 (C-2), 126.86 (C-4′, C-2′), 128.92 (C-1′, C-3′, C-5′).

*6-(4-Chlorophenyl)-3-phenylpyrazolo[1,5-a]pyrimidine* (**15**): Compound **3** (150 mg, 0.94 mmol) was dissolved in 7 mL of 10% AcOH/EtOH, and (*E*)-2-(4-chlorophenyl)-3-hydroxyacrylaldehyde (172 mg, 0.94 mmol) was added. The reaction was carried out as previously described for the synthesis of pyrazolopyrimidine derivatives to afford pyrimidine **15** as yellow crystals (229 mg, 0.75 mmol, yield: 78%) (Mp: 132 °C). **^1^H NMR** (CDCl_3_): 9.27 (1H, d, J 2.4 Hz, H-6), 9.15 (1H, d, J 2.4 Hz H-4), 8.78 (1H, s, H-1), 8.0 (2H, d, J 8.4 Hz, H-1′, H-5′), 7.69 (2H, d, J 8.4 Hz, H-1″, H-5″), 7.55 (2H, d, J 8.4 Hz, H-2″, H-4″), 7.48 (3H, t, J 7.8 Hz, H-2′, H-3′, H-4′). **^13^C NMR** (Acetone-d6): *δ* = 153.8 (C-6), 149.3 (C-4), 140.1 (C-3″), 133.81 (C-5), 130.15 (C-6″), 130.03 (C-2″, C-4”), 128.1 (C-4′, C-2′), 127.6 (C-1′, C-3′, C-5′), 101.12 (C-2).

*3-(4-Fluorophenyl)-6-(4-methoxyphenyl)pyrazolo[1,5-a]pyrimidine* (**17**): Compound **16** (150 mg, 0.85 mmol) was dissolved in 7 mL of 10% AcOH/EtOH, and the resulting mixture was reacted with 2-(-4-methoxyphenyl) malondialdehyde (151.5 mg, 0.85 mmol), yielding **17** in crude form. Purification with flash column chromatography using CH_2_Cl_2_ as the eluent provided **17** as yellow crystals (220 mg, 0.69 mmol, yield: 81%) (Mp: 175–176 °C). **^1^H NMR** (CDCl_3_): 8.71 (2H, d, J 8.7 Hz, H-4, H-6), 8.4 (1H, s, H-1), 7.80 (2H, m, H-1′, H-5′), 7.47 (2H, d, J 8.4 Hz, H-1″, H-5″), 7.09 (2H, t, J 9.0 Hz, H-2′, H-4′), 7.00 (2H, d, J 9.0 Hz, H-2″, H-4″), 3.92 (3H, OCH_3_) **^13^C NMR** (Acetone-d6): *δ* = 149.96 (C-4), 143.81 (C-1), 131.539 (C-6), 110.49 (C-2), 128.18 (C-1′, C-5′), 127.89 (C-1″, C-5″), 116.04 (C-2′, C-4′), 115.18 (C-2″, C-4″), 162.0 (C-3″), 122.56 (C-6″), 159.68 (C-6′), 55.85 (O-CH_3_).

### 2.3. General Procedure for the Preparation of Phenol Derivatives

To an ice-cold (0 °C) stirred solution of sodium hydride (60% dispersion in oil, 0.77 mmol) in 1 mL of anhydrous DMF, maintained under an argon atmosphere, a solution of phenol **5** (0.70 mmol) in 1 mL anhydrous DMF was added in portions over 10 min. After stirring for 30 min, a solution of the respective carbonyl chloride (1.05 mmol) in DMF (0.5 mL) was added. The mixture was left to reach room temperature and then heated gradually to 250 °C. Stirring was continued for an additional hour, after which the reaction was quenched with a saturated solution of NH_4_Cl and extracted with EtOAc (2 × 25 mL). The combined organic phases were washed with brine, dried over anhydrous MgSO_4_, and the solvent was removed under vacuum. Flash column chromatography purification of the resulting slurry using EtOAc/Hexane (1:1) as the eluant provided the derivatized end products in pure form.

*4-(3-Phenylpyrazolo[1,5-a]pyrimidin-6-yl)phenylpyrrolidine-1-carboxylate* (**10**): To a stirred solution (maintained at 0 °C with ice) of sodium hydride (60% dispersion in oil, 30.8 mg, 0.77 mmol) in 1 mL of anhydrous DMF, under argon atmosphere, a solution of compound **5** (200 mg, 0.70 mmol) in 1 mL of anhydrous DMF was added dropwise in a 10 min period. After stirring for 30 min, a solution of pyrrolidine carbonyl chloride (1.05 mmol, 0.12 mL) in DMF (0.5 mL) was added, and the mixture was gradually heated to 250 °C and stirred for an additional hour. The reaction was quenched with saturated solution of NH_4_Cl and extracted with EtOAc (2 × 25 mL). The combined organic phases were washed with brine and dried with anhydrous MgSO_4_, and the solvent was evaporated under vacuum. The resulting slurry was purified by flash column chromatography using EtOAc/Hexane (1:1), yielding **10** in the form of a yellow crystalline solid (200 mg, 0.52 mmol, yield: 68%), (Mp: 201 °C). **^1^H NMR** (CDCl_3_): 8.76 (2H, d, J 7.2 Hz, H-4, H-6), 8.49 (1H, s, H-1), 8.10 (2H, d, J 7.2 Hz, H-1′, H-5′), 7.63 (2H, d, J 6.5 Hz, H-1″, H-5″), 6.51 (2H, t, J 7.2 Hz, H-4′, H-2′), 7.31 (3H, m, H-3′, H-2″, H-4″), 3.65 (2H, s, H-1‴, H-4‴), 3.57 (2H, s, H-1‴, H-4‴), 2.00 (4H, m, H-2‴, H-3‴). **^13^C NMR** (Acetone-d6): *δ* = 149.16 (C-4), 143.25 (C-1), 132.18 (C-6), 128.87 (C-4′, C-2′), 126.57 (C-1′, C-5′), 127.86 (C-1″, C-5″), 126.71 (C-3′), 123.40 (C-2″, C-4″), 110.63 (C-2), 131.36 (C-6′), 151.89 (C-3″), 130.53 (C-6″), 46.55 (C-1‴, C-4‴), 25.80 and 25.01 (C-2‴, C-3‴).

*4-(3-Phenylpyrazolo[1,5-a]pyrimidin-6-yl)phenyl diethylcarbamate* (**11**): Compound **9** was synthesized following the above-described general procedure, using as substrate the diethylcarbamoyl chloride (1.05 mmol, 0.13 mL). Flash column chromatography purification (EtOAc/Hexane 5.0/5.0) provided pure **11** (200 mg, 0.54 mmol, yield: 70%) as pale yellow crystals (Mp: 190 °C). **^1^H NMR** (CDCl_3_): *δ* = 8.8 (m, 2H, H-4, H-6), 8.49 (s, 1H, H-1), 8.08 (d, J = 7.2 Hz, 2H, H-1′, H-5′), 7.63 (d, J = 8.4 Hz, 2H, H-1″, H-5″), 7.49 (t, J = 7.8 Hz, 2H, H-2′, H-4′), 7.30 (m, 3H, H-3′, H-2″, H-4″), 3.50 (dd, J 6.6 Hz, 4H, CH_2_CH_3_), 1.30 (dt, J 6.6 Hz, 6H, CH_2_CH_3_). **^13^C NMR** (CDCl_3_): *δ* = 149.1 (C-6), 144.21 (C-1), 132.24 (C-4), 110.46 (C-2), 131.21 (C-3, C-5, C-6″), 126.53 (C-1′, C-5′), 128.18 (C-1″, C-5″), 128.8 (C-2′, C-4′), 126.6 (C-3′), 123.0 (C-2″, C-4″), 152.26 (C-3″).

*4-(3-Phenylpyrazolo[1,5-a]pyrimidin-6-yl)phenyl piperidine-1-carboxylate* (**12**): Compound **12** was prepared, using the previously described procedure with substrate, a solution of piperidinecarbonyl chloride (1.05 mmol, 0.13 mL) in 0.5 mL anhydrous DMF. Purification by flash column chromatography (EtOAc/Hexane 1:1 as the eluent) yielded compound **12** (230 mg, 0.58 mmol, yield: 75%) as yellow crystalline needles (Mp: 177 °C). **1H NMR** (CDCl_3_): 8.85 (2H, q, J 3.6 Hz, H-4, H-6), 8.46 (1H, s, H-1), 8.03 (2H, d, J 7.2 Hz, H-1′, H-5′), 7.65 (2H, d, J 8.4 Hz, H-1″, H-5″), 7.53 (2H, t, J 7.8 Hz, H-2′, H-4′), 7.28 (3H, m, H-3′, H-2″, H-4″), 3.60 (6H, m, H-2‴, H-3‴, H-4‴), 1.7 (4H, m, H-1‴, H-5‴). **^13^C NMR** (Acetone-d6): δ = 149.35 (C-6), 143.09 (C-1), 131.85 (C-4), 128.76 (C-4′, C-2′), 127.95 (C-1″, C-5″), 126.51 (C-3′), 126.70 (C-1′, C-5′), 122.91 (C-2″, C-4″), 151.7 (C-3″), 130.82 (C-5), 110.897 (C-2), 131.97 (C-6′), 45.61 (C-2‴, C-3‴, C-4‴), 24.81 (C-1‴, C-5‴).

*4-(3-Phenylpyrazolo[1,5-a]pyrimidin-6-yl)phenyl dimethylcarbamate* (**8**): Compound **8** was prepared according to the above-described general procedure, using dimethylcarbamyl chloride (1.05 mmol, 0.1 mL). Flash column chromatography with EtOAc/Hexane, 7/3, furnished the anticipated carbamate derivative **8** as a yellow solid (180 mg, 0.50 mmol, yield: 71%), which was recrystallized from diethyl ether (Mp: 171 °C). **^1^H NMR** (CDCl_3_): *δ* = 8.85 (d, J = 8.4 Hz, 2H, H-2, H-3), 8.50 (s, 1H, H-1), 8.09 (dd, J = 7.2 Hz, 2H, H-1′, H-2′), 7.62 (d, J = 9.0 Hz, 2H, H-1″, H-2″), 7.50 (t, J = 9.0 Hz, 2H, ArH), 7.32 (m, 3H, ArH), 3.17 (s, 3H, -CH_3_), 3.08 (s, 3H, -CH_3_).

*4-(3-Phenylpyrazolo[1,5-a]pyrimidin-6-yl)phenyl diisopropylcarbamate* (**13**): Reaction of *N,N*-diisopropylcarbamyl chloride (1.05 mmol, 171.8 mg) yielded compound **13**, which was cleansed (column chromatographed, EtOAc/Hexane) to yield **13** as yellow crystals (200 mg, 0.48 mmol, yield: 69%), (Mp: 192 °C). **^1^H NMR** (CDCl_3_): 8.74 (2H, m, H-4, H-6), 8.390 (s, 1H, H-1), 7.99 (2H, d, J 7.2 Hz, H-1′, H-5′), 7.53 (2H, d, J 8.4Hz, H-1″, H-5″), 7.41 (2H, t, J 7.5 Hz, H-2′,H-4′), 7.23 (3H, d, J 8.4 Hz, H-2″, H-4″, H-3′), 3.41 (2H, q, J 6.6 Hz, CH(CH_3_)_2_), 1.25 (m, 12H, CH(CH_3_)_2_).

*4-(3-Phenylpyrazolo[1,5-a]pyrimidin-6-yl)phenyl methyl(phenyl)carbamate* (**6**): This molecule was obtained by reacting compound **5** (200 mg, 0.70 mmol) dissolved in 1 mL anhydrous DMF with a solution of *N*-methyl-*N*-phenylcarbamoyl chloride (1.05 mmol, 178.1 mg) in 1 mL anhydrous DMF. The resulting residue was separated by decantation and purified by flash column chromatography using EtOAc/Hexane (1:1), yielding **6** (270 mg, 0.64 mmol, yield: 83%) as yellow crystalline needles (Mp: 215 °C). **^1^H NMR** (CDCl_3_): 9.54 (1H, d, J 1.8 Hz, H-6), 9.07 (1H, d, J 2.4 Hz, H-4), 8.82 (1H, s, H-1), 8.19 (2H, d, J 7.2 Hz, H-1′, H-5′), 7.94 (2H, d, J 9.0 Hz, H-1″, H-5″), 7.38 (9H, m, ArH), 7.28 (3H, m, ArH H-3‴, H-2″, H-4″), 4.03 (3H, s, -NCH_3_).

*O-(4-(3-Phenylpyrazolo[1,5-a]pyrimidin-6-yl)phenyl)dimethylcarbamothioate* (**9**): Compound **9** was prepared following the general procedure for the synthesis of phenol derivatives, with dimethylthiocarbamoyl chloride (1.05 mmol, 129.8 mg) as the substrate. Purification with flash column chromatography (EtOAc/Hexane, 3/7) yielded the carbamothioate derivative as pale yellow fine crystals (170 mg, 0.45 mmol, yield: 65%) (Mp: 167–168 °C). **^1^H NMR** (CDCl_3_): *δ* = 8.87 (d, 2H, J = 6.6 Hz, H-4, H-6), 8.50 (s, 1H, H-1), 8.09 (d, J = 7.8 Hz, 2H, H-1′, H-5′), 7.67 (d, J = 8.4 Hz, 2H, H-1″, H-5″), 7.51 (t, J = 7.8 Hz, 2H, H-2″, H-4″), 7.29 (t, J = 8.4 Hz, 1H, H-3′), 7.28 (d, J = 8.0 Hz, 2H, H-2″, H-4″), 3.52 (s, 3H, -CH_3_), 3.42 (s, 3H, -CH_3_).

*4-(3-Phenylpyrazolo[1,5-a]pyrimidin-6-yl)phenyl diphenylcarbamate* (**7**): The same procedure using compound **5** (200 mg, 0.70 mmol) and diphenylcarbamoyl chloride (1.05 mmol, 243.3 mg) as substrates provided carbamate derivative **7** as a yellow solid (300 mg, 0.66 mmol, yield: 85%) (Mp: 189 °C). **^1^H NMR** (400 MHz, CDCl_3_): 8.83 (2H, d, J 8.8 Hz, H-4, H-6), 8.49 (1H, s, H-1), 8.09 (2H, d, J 8.2 Hz, H-1′, H-5′), 7.62 (2H, d, J 8.4 Hz, H-1″, H-5″), 7.5 (2H, t, J 7.5 Hz, H-2′, H-4′), 7.44–7.36 (13H, m, ArH).

*4-(4-Fluorophenyl)-1H-pyrazol-5-amine* (**16**): A similar procedure for the synthesis of amine **4** using 4-fluorophenylacetonitrile (2 mL, 16.7 mmol) and hydrazine (3.7 mL, 120 mmol) as substrates provided amine **16**. Flash column chromatography with pure CH_2_Cl_2_ as the eluent yielded **16** (1.8 g, 10.16 mmol) in the form of pale yellow crystals.

*4-(3-Phenylpyrazolo[1,5-a]pyrimidin-6-yl)phenol* (**5**): Cleavage of the methoxy group of compound **4** was achieved by dissolving 300 mg (0.99 mmol) in 10 mL of 50% AcOH/EtOH and refluxing the mixture (HBr, 48%) for 24 h at 300 °C. When the reaction end was verified by TLC, CH_2_Cl_2_ was added to afford a residue then purified by flash column chromatography with EtOAc:Hexane (3:7) as the eluent to provide phenol **5** as yellow crystalline needles (250 mg, 0.87 mmol, yield: 88%). NMR data were consistent with those in the literature.

*4,4′-(Pyrazolo[1,5-a]pyrimidine-3,6-diyl)diphenol* (**22**): A similar procedure was applied for the methoxy group cleavage of **21**. In particular, this compound (300 mg, 0.90 mmol) was dissolved in 10 mL of 50% AcOH/EtOH, and the mixture was refluxed with HBr (48%) for 10 h at 250 °C. When TLC indicated the end of the reaction, the resulting residue was purified by flash column chromatography using CH_2_Cl_2_ as the eluent providing diphenol **22** (250 mg, 0.87 mmol, yield: 97%) as yellow crystalline needles (Mp: 164 °C). **^1^H NMR** (Acetone-d6): 8.89 (s, 1H, H-6), 8.74 (s, 1H, H-4), 8.33 (s, 1H, H-1), 7.89 (2H, d, J 7.71 Hz, H-1′, H-5′), 7.55 (2H, d, J 7.70 Hz, H-1″, H-5″), 6.90 (2H, d, J 7.48 Hz, H-2″, H-4″), 6.81 (2H, d, J 7.91 Hz, H-2′, H-4′). **^13^C NMR** (Acetone-d6): *δ* = 148.95 (C-4), 142.46 (C-1), 131.55 (C-6), 109.8 (C-2), 127.44 (C-1′, C-5′), 128.44 (C-1″ και C-5″), 115.66 (C-2′, C-4′), 116.27 (C-2″, C-4″), 123.75 (C-6′), 123.66 (C-6″), 155.97 (C-3′), 157.76 (C-3″), 122.43 (C-3), 54.82 (OCH_3_).

*4-(3-(4-Fluorophenyl)pyrazolo[1,5-a]pyrimidin-6-yl)phenol* (**18**): Ether **17** (300 mg, 0.94 mmol) was dissolved in 10 mL 50% AcOH/EtOH and refluxed with HBr (48%) for 4 h at 250 °C, as described before. After completion of the reaction (confirmed by TLC), and subsequent workup, the end product **18** was obtained in pure form using column chromatography (Hexane/EtOAc 6:4) as a yellow solid (230 mg, 0.75 mmol, yield: 80%) (Mp: 159 °C). **^1^H NMR** (Acetone-d6): 9.0 (1H, s, H-6), 8.9 (s, 1H, H-4), 8.62 (1H, s, H-1), 8.8 (1H, s, OH), 8.25 (2H, m, H-1′, H-5′), 7.72 (2H, d, J 9.0 Hz, H-1″, H-5″), 7.25 (2H, t, J 9.0 Hz, H-2′, H-4′), 7.05 (2H, d, J 8.4 Hz, H-2″, H-4″). **^13^C NMR** (Acetone-d6): *δ* = 149.40 (C-4), 142.19 (C-1), 131.202 (C-6), 108.82 (C-2), 129.79 (C-3), 127.63 (C-1′, C-5′), 128.1 (C-1″, C-5″), 115.59 (C-2′, C-4′), 116.04 (C-2″, C-4″), 157.76 (C-3″), 147.7 (C-3′), 123.68 (C-6″), 159.68 (C-6′).

*6-(4-Chlorophenyl)-3-(4-fluorophenyl)pyrazolo[1,5-a]pyrimidine* (**19**): Fluoro derivative **16** (150 mg, 0.85 mmol) was dissolved in 7 mL 10% AcOH/EtOH and 2-(4-chlorophenyl)malondialdehyde (155.2 mg, 0.85 mmol) was added. The mixture was treated as above, providing **19** (after flash column chromatography, with Hexane/EtOAc 6:4) as a yellow solid (220 mg, 0.68 mmol, yield: 80%) (Mp: 178 °C). **^1^H NMR** (CDCl_3_): *δ* = 8.76 (d, J = 2.31 Hz, 1H, H-6), 8.72 (d, J = 2.31 Hz, 1H, H-4), 8.36 (s, 1H, H-1), 7.95 (m, 2H, H-1′, H-5′), 7.46 (m, 4H, H-1″, H-2″, H-4″, H-5″), 7.10 (t, J = 8.74 Hz, 2H, H-2′, H-4′). **^13^C NMR** (CDCl_3_): *δ* = 148.58 (C-6), 143.91 (C-1), 132.34 (C-4), 110.68 (C-2), 132.51 (C-3, C-5, C-6″), 127.85 (C-1′, C-5′), 127.97 (C-1″, C-5″), 115.34 (C-2′, C-4′), 132.0 (C-2″, C-4″, C-6″), 135.37 (C-3″), 162.02 (C-3′).

### 2.4. Molecular Docking Calculations

All molecular docking calculations were performed via the ‘RunRxock’ Asclepios KNIME node, which utilizes the RxDock^1^ engine as implemented in the Enalos Asclepios KNIME nodes [25,26].

#### 2.4.1. Protein Structure Preparation

**Figure 2:**
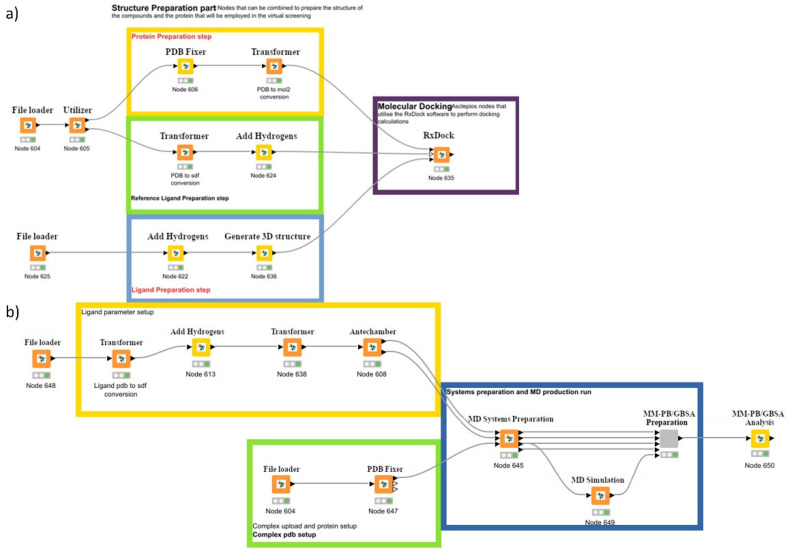
The Asclepios KNIME pipeline highlighting (**a**) the docking process and (**b**) the MM-GBSA refinement process implemented in the present work.

The crystal structures of the (a) AMP-activated protein kinase alpha 2 subunit (PDB ID: 3AQV) [27], (b) Activin receptor type-1 kinase domain (ALK2) (PDB ID: 3Q4U, chain C) [28], (c) DKK-1 domain (PDB ID: 3S2K, chain C) [29], (d) transforming growth factor β (TGFβ) type I receptor (PDB ID: 2WOT) [30], and (e) ABCG2 transporter (PDB ID: 6VXI) [31] were initially retrieved from the protein data bank. Then, protein molecules were curated with the “Asclepios Fixer” node and the “Protein structure preparation” branch of the Enalos Asclepios KNIME pipeline (Figure 2a).

#### 2.4.2. Ligand Structure Preparation

All ligands were prepared using the “Ligand structure preparation” of the Enalos Asclepios KNIME pipeline. The protonation states of the compounds were considered at physiological pH (7.4) and were determined using the ‘Add Hydrogens’ node, which involved adding missing hydrogen atoms via Open Babel [32]. Prior to docking, all compounds were subjected to geometry optimization with the ‘AsclepiosGenerate3Dcoords’ node using the Merck molecular force field (MMFF94) [33,34,35,36,37].

#### 2.4.3. Molecular Docking Setup

The binding cavities for the different proteins were determined using the reference ligand method with a radius of 8 Å around the bound inhibitor, and a small sphere radius of 1.0 Å. The only exception was the DKK-1 protein where the binding sites were defined using the two-spheres method implemented in the RxDock Asclepios node. Two possible binding sites were identified with the aid of the DoGSiteScorer web server for automatic binding site prediction [38]. The RbtCavityGridSF scoring function was employed, setting a “cavity restrain function” with a weight of 1 to prevent the ligand from exiting the docking site. The maximum number of acceptable cavities was set to 1, and the minimum acceptable cavity volume was 100 Å^3^. Mapping of the binding sites was conducted using a grid resolution of 0.5 Å. Once the active site in each protein was designated, a grid file was generated, and each ligand was docked using a 100 runs per ligand RxDock job. Molecular docking solutions were then sorted based on their corresponding intermolecular energy scores, as this better reproduces the crystallographic structures of the reference ligands considered. It has been shown in the literature that even though shorting should ideally be based on their total score, which includes intramolecular energy, the inclusion of an intramolecular term often introduces significant errors, thereby compromising predictive accuracy [39,40].

#### 2.4.4. MM-GABS Docking Refinement

The best docking poses for the most prominent compound candidate were refined using the MM-PBS A protocol as implemented in the Enalos Asclepios KNIME pipeline (Figure 2b). The best docking pose of the compound was loaded in the workflow, and the ligand was prepared using the ‘Add Hydrogens’ node. The ‘Transformer’ and ‘Antechamber’ nodes were employed to build ligand parameters, while the ‘MD Systems Preparation’ node was employed to build the complex parameters using the tleap module of AmberTools23 [41]. Minimization and equilibration of the complexes were performed using the OpenMM software [42]. Equilibration was performed for 4 ns at 300 K and pressure of 1 atm. Soft positional restraints were imposed on the complex for the first 3 ns and gradually abolished with the last 1 ns of equilibration performed, with no positional restraints. The complexes were subjected to MM-GBSA calculations for estimating the binding enthalpy of the system. MM-GBSA analysis was performed through the implementation of AmberTools23 in the respective MM-PB/GBSA analysis node from the Enalos Asclepios KNIME nodes.

### 2.5. Cell Proliferation Assays

Cell lines (endothelial and tumor) were obtained from suppliers, treated, and counted (Coulter counter, Analis, Belgium), as reported in a previously published work by our group [43].

#### 2.5.1. Endothelial Cells

Bovine aortic endothelial cells (BAECs) and human dermal microvascular endothelial cells (HMEC-1) were seeded in 48-well plates at 10,000 cells/well and 20,000 cells/well, respectively. After 24 h, 5-fold dilutions of the compounds were added (namely 100-20-4-1-0.2 μM). The cells were permitted to proliferate for 3 days (or 4 days for HMEC-1) in the presence of the compounds, then trypsinized, and counted. As a positive control, the anticancer drug daunorubicin was used.

#### 2.5.2. Tumor Cells

Human cervical carcinoma (HeLa) cells were seeded in 96-well plates at 15,000 cells/well with different concentrations of the compounds. After 4 days of incubation, the cells were trypsinized and counted. Suspension cells (Mouse leukemia L1210 and human lymphoid CEM cells) were seeded in 96-well plates at 60,000 cells/well in the presence of diverse concentrations of the molecules (see range above). L1210 and CEM cells were allowed to proliferate for 48 h or 96 h, respectively, and then counted. As a positive control, the anticancer drug melphalan was used.

The 50% inhibitory concentration (IC50) was delineated as the compound’s concentration required to reduce cell proliferation by 50%. To distinguish live from dead cells, flow cytometric analysis was conducted on a BD FACSCanto II flow cytometer (BD Biosciences, New Jersey, USA) [44]. Data were processed using the FlowJo software (Tree Star, Ashland, OR, USA). Statistical analysis was conducted using XLSTAT 2022.1.1 software (Microsoft Excel version 16.94 209).

## 3. Results and Discussion

### 3.1. Synthesis

Five precursors and ten novel DOS derivatives were synthesized from the phenolic pyrazolopyrimidine **5**, which served as the common key intermediate. The latter was prepared according to a synthetic procedure proposed by Daniels et al. [45], which was modified at the step concerning the formation of compound **3**. Herein, we implemented this reaction under acidic conditions by adding AcOH, since in our hands, the reaction performance under non-acidic conditions (as described by Daniels) was not successful and did not yield the desired pyrazolamine **3** molecule. Instead, under these conditions, we recovered the unreacted (Z)-3-(dimethylamino)-2-phenylacrylonitrile. The need to employ acidic conditions was reinforced by relevant findings from other groups focusing on the preparation of 5-amino-pyrazoles [for an informative review, see reference [46]]. Indicatively, Marinozzi et al. added 12 M HCl at their last synthetic step in order to obtain the desired aryl-5-amino pyrazoles [47], possibly because the lack of a temperature controller in the microwave system does not permit the reproductive achievement of a 140 °C temperature. However, verification of this assumption needs further experimentation.

**Figure 3:**
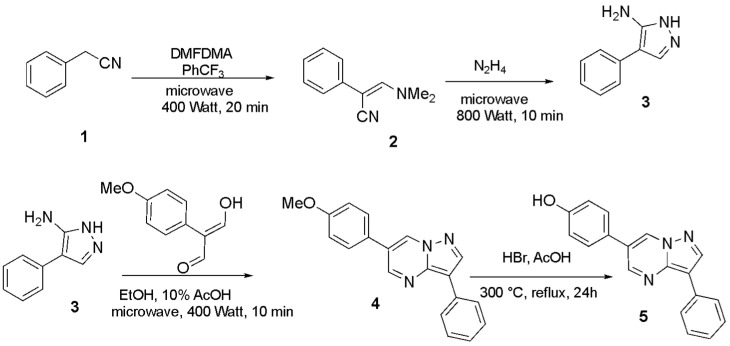
Preparation of key intermediate **5**.

Synthesis of the key intermediate pyrazolo[1,5-*a*]pyrimidine **5** was accomplished in accordance with the synthetic sequence depicted in Figure 3. In particular, the reaction of phenylacetonitrile substrate with dimethyformamide dimethylacetal (DMFDMA) provided the intermediate **2**, which was cyclized in the presence of hydrazine to 2-amino-1H-pyrazole **3**. The latter was condensed with several malondialdehydes in acetic acid and ethanol under heating (conventional or microwave-assisted conditions), yielding the pyrazolo[1,5-*a*]pyrimidine derivative **4**, which was further dealkylated by reacting with hydrobromic acid in acetic acid to furnish target compound **5**. It must be noted that the formation of pyrazolopyrimidine **4** proceeded through double imine condensation in acidic media (for the initial acidic activation of carbonyl moieties at both steps, as well as activation for the dehydration processes, see the mechanism in Figure S47).

**Figure 4:**
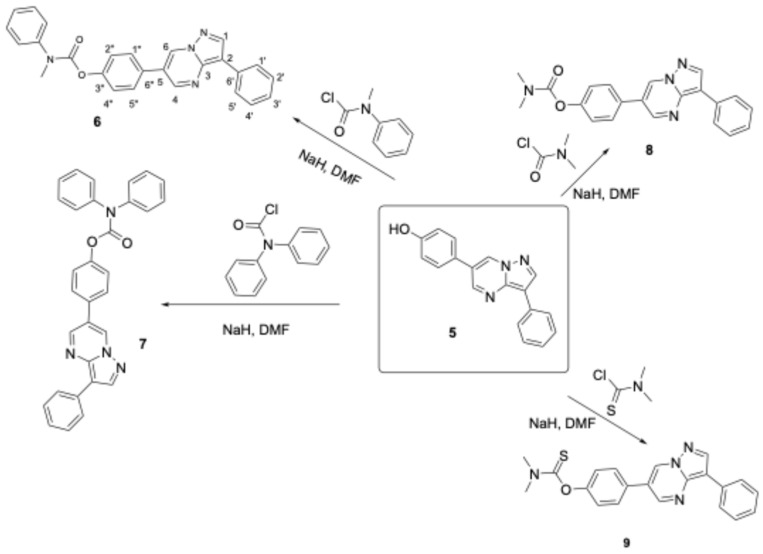
The new DOS derivatives **6**–**9**.

**Figure 5:**
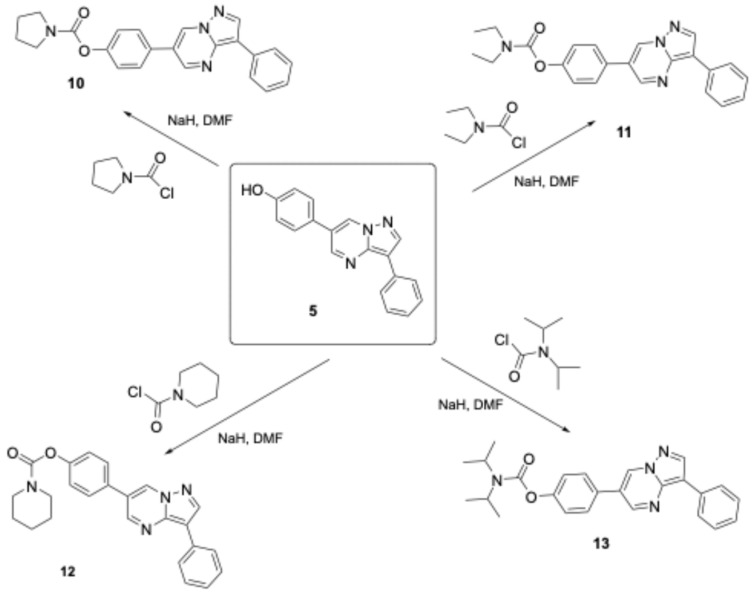
The new DOS derivatives **10**–**13**.

**Figure 6:**
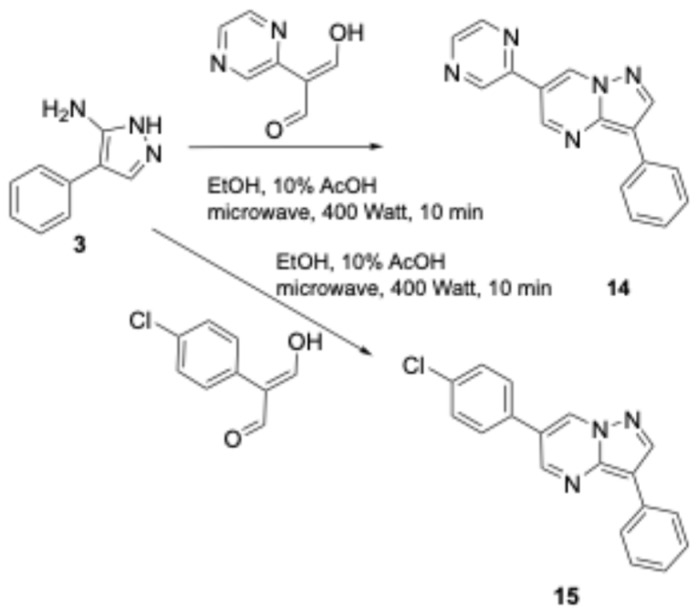
The new DOS derivatives **14**, **15**.

**Figure 7:**
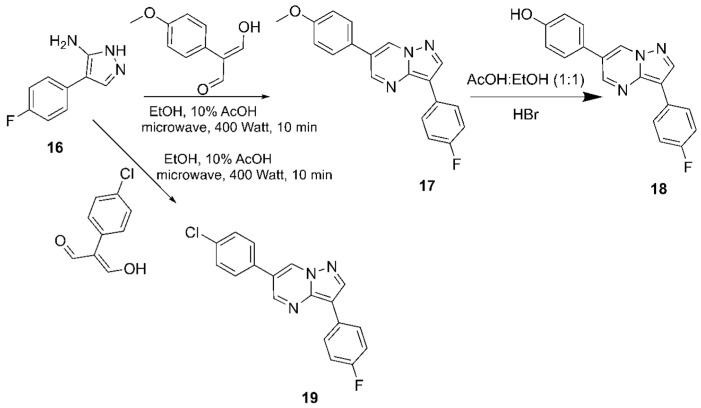
The new DOS derivatives **17**–**19**.

**Figure 8:**
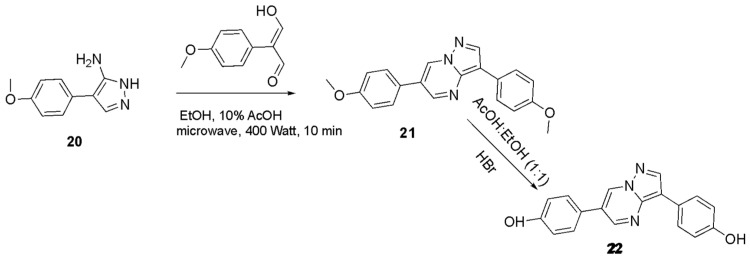
The new DOS derivatives **21**, **22**.

**Figure 9:**
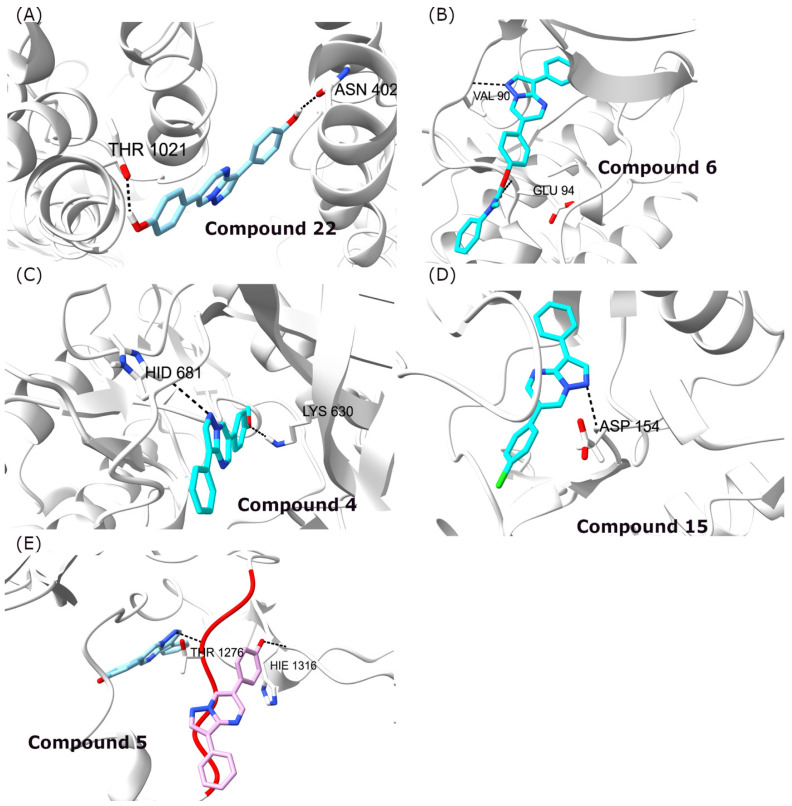
Docking conformations of the best scoring compounds in the different protein targets. (**A**) Compound **22** in complex with the ABCG2 receptor, (**B**) compound **6** in complex with the AMPK receptor, (**C**) compound **4** in complex with the Activin receptor type-1 kinase domain, (**D**) compound **15** in complex with the TGFβ type I receptor, and (**E**) compound **5** (cyan and pink) in complex with the Dickkopf-1 protein at the two possible sites considered.

Attempts to cleave the methoxy groups with the addition of HBr and AcOH and the utilization of microwaves were not satisfying since they provided only a 20–30% yield of the desired phenol when 50% AcOH/EtOH was applied at 1000 Watts for 15 min. In contrast, refluxing the reaction mixture with conventional heating furnished the deprotected compounds in high yields (80–90%). Alternatively, attempting to deprotect these ethers by adding BBr_3_ provided low yields (25–30%), except for compound **21**, which underwent O-demethylation smoothly with a yield of 65–70%. In contrast, for the synthesis of the derivatized products, a mixture of phenol **5** with sodium hydrazide was reacted with various carbamoyl chlorides in DMF solvent, providing compounds **6**–**13** in satisfactory yields (65–85%, Figure 4 and Figure 5). Condensation of compound **3**, with several dialdehydes, yielded **14**, **15**, **17**, and **19** (yields in the range of 75–81%, Figure 6 and Figure 7), and a similar reaction of 1H-pyrazol-5-amine derivative **20** provided pyrazolo[1,5-*a*]pyrimidine **21**, almost quantitatively (Figure 8). Finally, the deprotection of compound **21** using the above-mentioned conditions provided the diphenolic compound **22** in 97% yield (Figure 9).

### 3.2. Proliferation of Endothelial and Tumor Cell Lines

It is noteworthy that the molecular modeling preceding the chemical synthesis showed encouraging binding affinities to different protein targets, in particular for compounds **6**, **18**, and **22**. In Table 1, the inhibitory effects of compounds on the proliferation of BAECs and HMEC-1 cells are presented, indicating that among the compounds investigated, only **22** and **6** inhibited endothelial cell proliferation in the lower micromolar range. Diphenol **22** was active in both cell lines tested, while compound **6** was only active in BAECs. Finally, it is apparent that the removal of the methoxy group of compound **21** (an inactive molecule) led to the discovery of **22**, which is a promising bioactive compound that corroborates the molecular modeling findings.

With respect to the activity of synthesized compounds against cancer cell lines (see Table 2), compound **22** was found to inhibit the proliferation of the L1210 and CEM leukemic cell lines in the lower micromolar range and was inactive against Hela cervical carcinoma cells. This result points to a specific action of this compound on a target shared between BAECs, HMEC-1, and L1210 cells, but is not present or important in HeLa cells. However, the observed cytotoxicity toward several normal and tumor cell lines sets a limitation to the further exploitation of this compound. Furthermore, phenol **18** was also determined to substantially inhibit the proliferation of the CEM and HeLa cell lines, indicating that the OH-group substitution with a fluorine atom seems to be the driving force behind the selectivity of compound **18** towards specific cell lines and its substantial activity against CEM cells, though it did not exhibit an optimum docking score. Overall, kinase-targeted cancer therapies are at the forefront of the development of new anti-cancer agents and serve as a promising starting point for the synthesis of bioactive molecules.

**Table 2:** Inhibitory effects of compounds on the proliferation of L1210, CEM, and HeLa cells.

IC50 (μM)			
Compound	L1210	CEM	HeLa
**4**	>250	>250	20 ± 5
**6**	99 ± 4	19 ± 11	14 ± 3
**7**	≥250	145± 35	32 ± 13
**8**	-	54 ± 16	>100
**10**	>250	84 ± 8	>250
**11**	>250	>250	>250
**12**	-	54 ± 26	>100
**13**	-	46 ± 3	>100
**14**	>250	>250	≥250
**15**	>100	36 ± 6	≥250
**17**	88 ± 15	82 ± 19	≥100
**18**	-	6.1 ± 1.5	14 ± 0
**19**	-	>100	>100
**21**	>250	>250	>250
**22**	8.6 ± 5.7	4.2 ± 3.6	198 ± 73
melphalan	2.8 ± 1.2	1.6 ± 0.5	3.5 ± 0.4

Within the tested concentration range and the allowed proliferation time points, the % viability was considered acceptable (see indicative cell viability diagram for compound **22** (for HMEC-1 cells) in Figure S48). A non-parametric Wilcoxon test was used to evaluate differences, and in all cases, the probability (*p*) was <0.05.

### 3.3. Docking Simulations

The results of the docking simulations are summarized in Table 3 and Table S1, indicating that the analysis of the docking scores does not reveal any common pattern across the various ligands. To validate the docking protocol, the crystallographic ligands were redocked, and the resulting poses reproduced the experimental binding orientations (Table S2, Figure S49) [48]. Moreover, the results suggest that there was no single ligand exhibiting stronger binding to all protein targets compared to the other derivatives. Specifically, compound **22** was determined as the top-scoring derivative for the ABCG2 transported, while compound **6** was found to bind tightly to AMPK (Table 3). In contrast, the best scoring hits were achieved by compound **4** for the ACVR1, compound **5** for the DKK-1, and compound **15** for the TGFβ type I receptor (Figure 9 and Table 3). The lack of a specific pattern in the derivatives studied can be rationalized considering the differences among various protein targets, which have all been identified as potential drug targets. Specifically, AMPK, ACVR1, and TGFβ type I receptors are implicated in BMP pathways. In this respect, DOS and its derivatives have been designed to target type I BMP receptors that are active in receptor-like kinases, as well as in non-BMP signaling pathways (including AMP-kinase and receptor tyrosine kinases involved in PDGF and VEGF signaling) [12,49]. Furthermore, dorsomorphin has been implicated in the inhibition of the Dickkopf-1 protein in breast cancer cell lines [50]. All these proteins have been reported to be targeted by inhibitors similar to DOS; therefore, the design of compounds based on this template molecule is promising for the development of new anti-cancer agents with improved activity. The only protein not directly associated with signaling is the ABCG2 transporter. It must be noted that ABC-type transporters have been implicated in the efflux of various compound from cells by ATP hydrolysis. This function and the subsequent overexpression of ABCG2 in cancer cells is one of the main obstacles in treating chemotherapy-resistant cancers [51]. Consequently, transporter inhibition may improve the response of patients to chemotherapy.

Based on the analysis of protein targets and the respective docking results, the top-scoring compounds create hydrogen bond interactions that anchor the molecules inside the binding site (Figure 9A–C). The hydrogen bonds are formed in the opposite sides of the ligand, allowing their better positioning and explaining their increased binding affinity. For the complex of compound **15** with the TGFβ type I receptor (Figure 9D), only one hydrogen bond was observed with Asp154, which anchors the ligand inside the binding site. In contrast, for the compound **5** complex with DKK-1 protein, two potential positions for binding the ligand were determined (Figure 9E). Since DKK-1 interacts with different proteins and not with small molecules inside the cell, two potential docking sites were investigated, both divided by the red-colored unstructured region (Figure 9E). Blocking of these sites interrupts the interaction of DKK-1 with other proteins, preventing its function in cancer cells. In contrast, compound **5** binds with a similar score in both regions (−27.658 and −24.182 kcal mol^−1^, Table S1) and creates hydrogen bond interactions with Thr1276 (Figure 9E, left) and His1316 (Figure 9E, right), respectively.

#### MM-GBSA Docking Refinement

**Figure 10:**
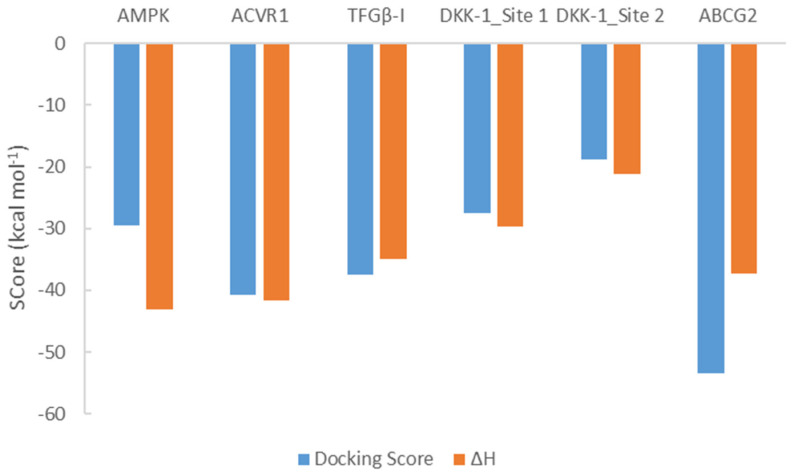
Docking scores and MM-GBSA calculated binding enthalpies for the complexes of compound **22** with all the proteins studied in this work.

Based on the docking and experimental results, compound **22** presented tight binding affinities in all targets. To further refine the results, the complexes were subjected to minimization and equilibration (described in Section 2.4.4). The calculated binding enthalpies (Δ*H*) highlight the improved binding of compound **22** in all proteins employed in this study. As presented in Figure 10 and Table S3, the refined values show that the dynamic nature of the binding process is better captured via MM-GBSA analysis. It is important to note that in the MM-GBSA calculations, the solvation effect is also taken into account, and this may explain the difference observed between the docking score and the enthalpy for the complexes of compound **22** with AMPK and the ABCG2 transporter (Table 4 and Table S3). The most important observation across all complexes is that the main contribution to binding derives from the van der Waals interactions, while the electrostatic contributions to binding appear to be the lowest in DKK-1 complexes (Table S3).

## 4. Conclusions

Several novel pyrazolo[1,5-*a*]pyrimidine derivatives (dorsomorphin analogs) were synthesized, and their inhibitory effects on the proliferation of murine leukemia cells (L1210), human T-lymphocyte cells (CEM), human cervix carcinoma cells (HeLa), and endothelial cells (human dermal microvascular (HMEC-1) cells and bovine aortic endothelial cells (BAECs)) were evaluated. Among these compounds, diphenol derivative **22** was unveiled as a promising lead molecule exhibiting bioactivity determined as IC_50_ values below 9 μM with the exemption of HeLa cells. In the same context, the carbamate analog **6** displayed a selective inhibitory activity against BAECs at the low micromolar range. The molecular docking calculations have revealed the potentials of these derivatives to act as potent inhibitors of different protein targets crucial for the development of cancer cells and their survival. Overall, among the nineteen novel derivatives synthesized and tested herein, compounds **6**, **18**, and **22** displayed the most promising results in impairing cell proliferation. This finding can be rationalized considering their favorable binding affinity towards the different protein targets. In particular, compound **22** exhibited tight binding against all protein targets studied, being the most potent inhibitor of ABCG2 receptor and the second-best scoring compound for AMPK, DKK-1, and the TGFβ type I receptors. Similarly, compound **6** showed a high binding affinity for all targets except the DKK-1 protein. The molecular docking simulations indicated that these derivatives display increased binding affinities for most protein targets studied, suggesting their potential to act as potent anti-cancer agents by targeting various proteins and inhibiting cancer cell proliferation.

## Figures and Tables

**Table 1:** Inhibitory effects of compounds on the proliferation of HMEC-1 cells and BAECs.

	IC50 (μM) *	
Compound	BAECs	HMEC-1
**4**	>100	>100
**6**	5.8 ± 2.2	87 ± 22
**7**	>100	39 ± 10
**8**	>100	>100
**10**	>100	>100
**11**	>100	73 ± 24
**12**	>100	>100
**13**	>100	>100
**14**	>100	>100
**15**	>100	68 ± 27
**17**	>100	78 ± 19
**18**	>100	39 ± 24
**19**	>100	>100
**21**	>100	≥100
**22**	2.4 ± 0.2	5.9 ± 2.3
daunorubicin	-	0.81 ± 0.11

* 50% inhibitory concentration, HMEC-1: human microvascular endothelial cell line, BAECs: bovine aortic endothelial cells.

**Table 3:** Docking scores (in kcal mol^−1^) of the compounds investigated in the selected protein targets.

Compound	Protein Target				
	AMPK ^a^	ACVR1 ^b^	TFGβ-I ^d^	DKK-1 ^c^	ABCG2 ^e^
**4**	−27.976	**−43.794** **^f^**	−37.201	−20.348	−45.498
**5**	−27.623	−38.547	−36.933	**−27.658**	−52.538
**6**	**−31.777**	−40.954	−33.149	−0.971	−32.275
**7**	−26.546	−42.602	−34.484	0.546	−50.264
**8**	−26.020	−42.451	−35.974	−6.075	−42.873
**9**	−24.430	−39.944	−35.391	0.592	−50.975
**10**	−25.148	−41.518	−27.105	−2.742	−34.507
**11**	−24.027	−41.495	−32.108	3.463	−47.188
**12**	−23.569	−36.545	−24.367	−3.132	−46.543
**13**	−22.790	−38.945	−28.283	−1.172	−37.762
**14**	−25.122	−41.916	−36.334	−24.274	−43.522
**15**	−26.114	−42.708	**−38.373**	−21.138	−37.493
**16**	−21.827	−39.005	−36.006	−19.335	42.792
**17**	−23.771	−39.127	−33.088	−16.306	−44.532
**18**	−26.595	−38.953	−29.526	−26.234	−52.600
**19**	−24.365	−41.745	−31.568	−21.506	−47.165
**20**	−25.066	−41.668	−29.653	−24.186	−43.582
**21**	−28.133	−42.980	−31.785	−14.373	−42.425
**22**	−29.522	−40.744	−37.390	−27.449	**−53.459**

^a^ AMP-activated protein kinase alpha 2 subunit, ^b^ Activin receptor type-1 kinase domain (ALK2), ^c^ Dickkopf-1 domain, ^d^ TGFβ type I receptor, ^e^ ABCG2 transporter, ^f^ in bold: best docking score.

**Table 4:** Docking scores and binding enthalpy calculated (MM-GBSA) for the complexes of compound **22** in the selected protein targets. All values are in kcal mol^−1^.

Protein Target	Compound 22	
	Docking Score	Δ*H* ^f^
**AMPK ^a^**	−29.52	−43.13 (2.96)
**ACVR1** ^b^	−40.74	−41.68 (2.82)
**TFGβ-I** ^d^	−37.39	−34.99 (2.24)
**DKK-1** ^c^		
Site 1	−27.45	−29.69 (1.88)
Site 2	−18.72	−21.11 (5.49)
**ABCG2** ^e^	−53.46	−37.21 (3.45)

^a^ AMP-activated protein kinase alpha 2 subunit, ^b^ Activin receptor type-1 kinase domain (ALK2), ^c^ Dickkopf-1 domain, ^d^ TGFβ type I receptor, ^e^ ABCG2 transporter, ^f^ standard deviation for the MM-GBSA calculations.

## Data Availability

All data are presented in this manuscript and the Supplementary Materials.

## Supplementary Materials

The following supporting information can be downloaded at: https://www.mdpi.com/article/10.3390/biom16010145/s1, Table S1: Docking scores (in kcal mol^−1^) of the compounds investigated in the pockets of DKK-1 predicted by DoGSiteScorer; Table S2: Docking scores (in kcal mol^−1^) of the reference ligands in the selected protein targets; Table S3: Decomposition of the enthalpy scores in all complexes of compound **22**. In parentheses are the standard deviations for each score. All values are in kcal mol^−1^; Figures S1–S46: NMR of investigated compounds; Figure S47: Mechanism of formation of pyrazolopyrimidine 4; Figure S48: Cell viability (%) diagram for diphenol 22 for HMEC-1 cells; Figure S49: Calculated conformations of the reference ligands in the different protein targets. (A) compound TAK in complex with the AMPK receptor, (B) compound ZZG in complex with the ACVR1 kinase domain, and (C) compound 15 in complex with the TGFβ type I receptor and (D) compound MIX in complex with the ABCG2 transporter. Crystallographic conformations are in black color. RMSDs between the predicted and crystallographic conformations were calculated by means of the LigRMSD server ^1^ and were found to be 1.18 Å, 0.90 Å, 0.57 Å, and 2.19 Å, respectively [48].

## Abbreviations

The following abbreviations are used in this manuscript:

BMP	bone morphogenetic protein
L1210	murine leukemia cells
CEM	human T-lymphocyte cells
HeLa	human cervix carcinoma cells
HMEC-1	human dermal microvascular
BAECs	bovine aortic endothelial cells
DOS	dorsomorphin dihydrochloride
