# Supplementary material for: Novel Dorsomorphin Derivatives: Molecular Modeling, Synthesis, and Bioactivity Evaluation
Source: Biomolecules. 2026 Jan 14;16(1):145. doi: 10.3390/biom16010145 (PMC12839441; doi:10.3390/biom16010145)
Supplement: Supplementary file 1 [file biomolecules-16-00145-s001.zip › biomolecules-4019674-supplementary.pdf]

Supplementary material

Novel Dorsomorphin Derivatives: Molecular Modeling, Synthesis and Evaluation of Bioactivity

Evangelia N. Tzanetou [1,†], Sandra Liekens [2], Konstantinos M. Kasiotis [1,†], Nikolas Fokialakis [3], Nikolaos Tsafantakis [3], Raul SanMartin [4], Haralampos Tzoupis [5], Konstantinos D. Papavasileiou [5,6,7], Antreas Afantitis [5,6,7,8] and Serkos A. Haroutounian [1,*]

Content

Table S1: Docking scores (in kcal mol^{-1}) of the compounds investigated in the pockets of DKK-1 predicted by DoGSiteScorer.

Compound	DKK-1	
	Pocket 1	Pocket 2
4	-20.348	-20.199
5	**-27.658**	**-24.182**
6	-0.971	-7.532
7	0.546	0.581
8	-6.075	-12.453
9	0.592	-17.058
10	-2.742	-16.135
11	3.463	-12.939
12	-3.132	-12.997
13	-1.172	0.699
14	-24.274	-17.263
15	-21.138	-18.475
16	-19.335	-23.120
17	-16.306	-19.451
18	-26.234	-17.858
19	-21.506	-19.982
20	-24.186	-21.311
21	-14.373	-20.197
22	-27.449	-18.718

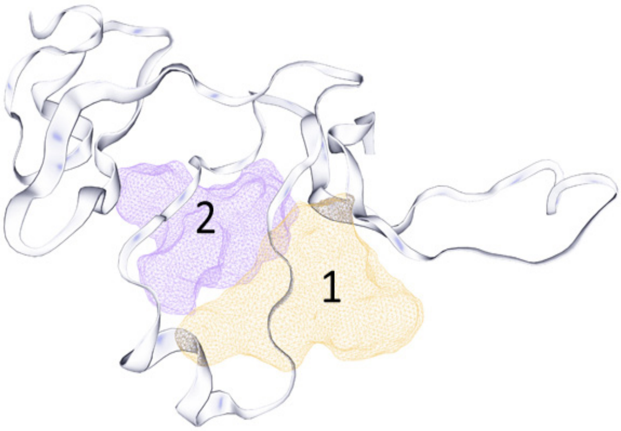

Table S2: Docking scores (in kcal mol^{-1}) of the reference ligands in the selected protein targets.

Compound	Protein target				
	AMPK[a]	ACVR1[b]	DKK-1[c]	TGFβ-I[d]	ABCG2[e]
TAK	-27.976	–			
LDN		-49.754			
–			–		
ZZG				-44.879	

MIX					-57.815

[a]AMP-activated protein kinase alpha 2 subunit
[b]Activin receptor type-1 kinase domain (ALK2)
[c]Dickkopf-1 domain
[d]TGFβ type I receptor
[e]ABCG2 transporter

Table S3: Decomposition of the enthalpy scores in all complexes of compound **22**. In parentheses are the standard deviations for each score. All values are in kcal mol^{-1}.

**Protein Target**	**Compound 22**				
	**vdW**	**Elec**	E_{GB}	E_{solv}	***H***
**AMPK**	-41.34 (2.71)	-33.13 (4.60)	36.69 (3.51)	31.34 (3.47)	-43.13 (2.96)
**ACVR1**	-39.52 (2.99)	-32.58 (4.80)	35.49 (2.74)	30.41 (2.67)	-41.68 (2.82)
**TFGβ-I**	-37.42 (1.68)	-21.14 (4.81)	28.73 (2.75)	23.58 (2.71)	-34.99 (2.24)
**DKK-1**					
Site 1	-32.51 (1.86)	-16.97 (2.86)	23.81 (2.54)	19.79 (2.50)	-29.69 (1.88)
Site 2	-26.59 (5.45)	-5.70 (3.98)	14.12 (3.85)	11.17 (3.56)	-21.11 (5.49)
**ABCG2**	-38.90 (1.90)	-20.85 (5.34)	27.52 (3.32)	22.55 (3.18)	-37.2 (3.45)

Figures S1-S46: NMR of investigated compounds

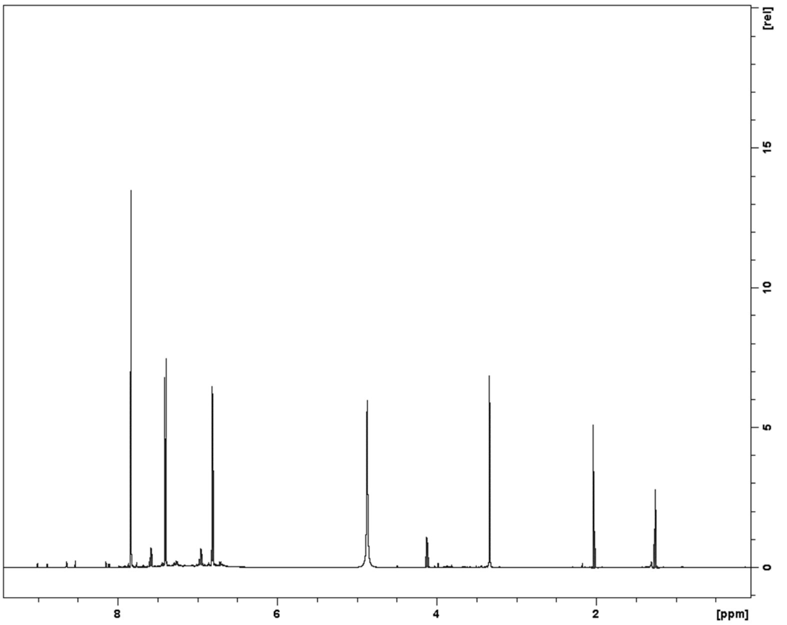

Figure S1. ^{1}H-NMR of compound 5

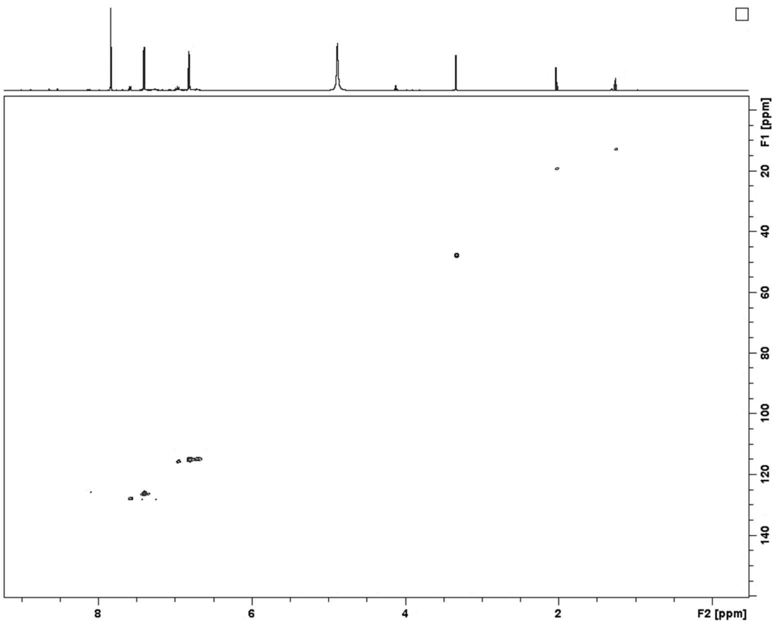

Figure S2. (^{1}H,^{13}C)-HSQC of compound 5

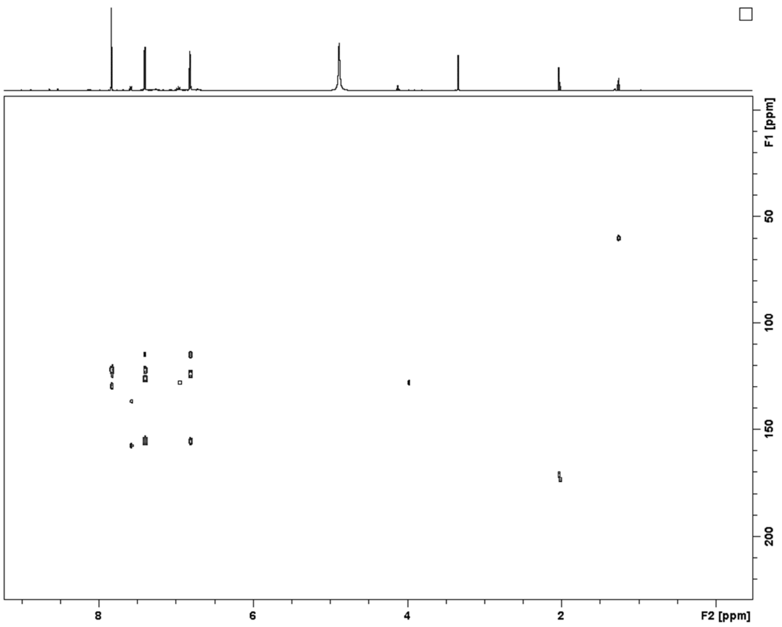

Figure S3. (^{1}H,^{13}C)-HMBC of compound 5

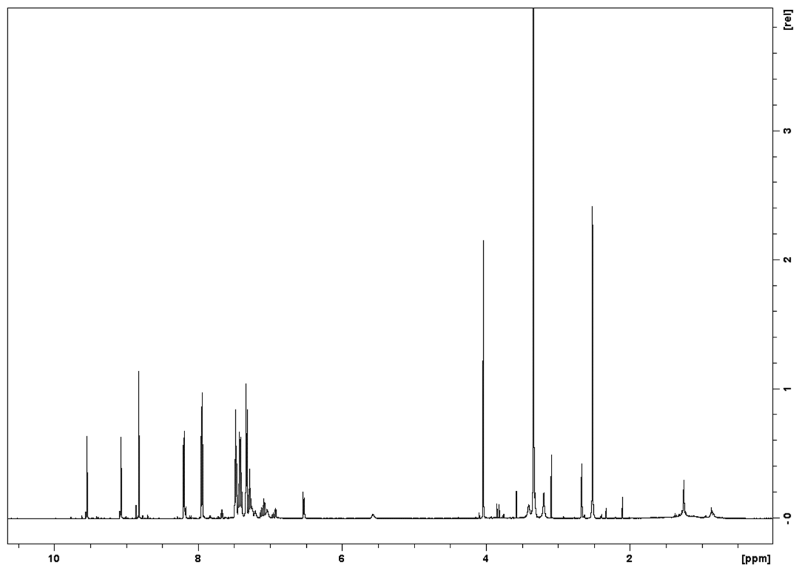

Figure S4. ^{1}H-NMR of compound 6

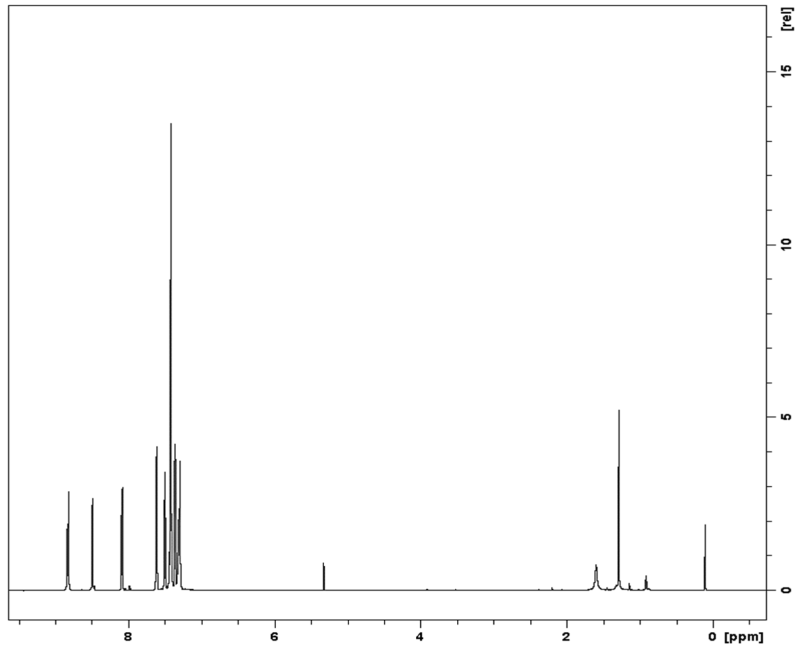

Figure S5. ^{1}H-NMR of compound 7

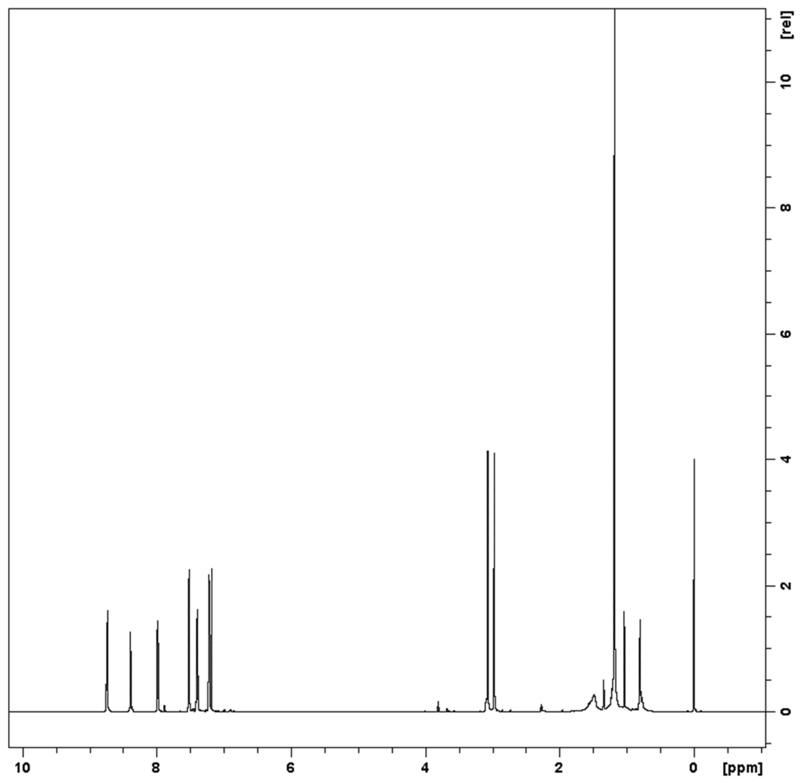

Figure S6. ^{1}H-NMR of compound 8

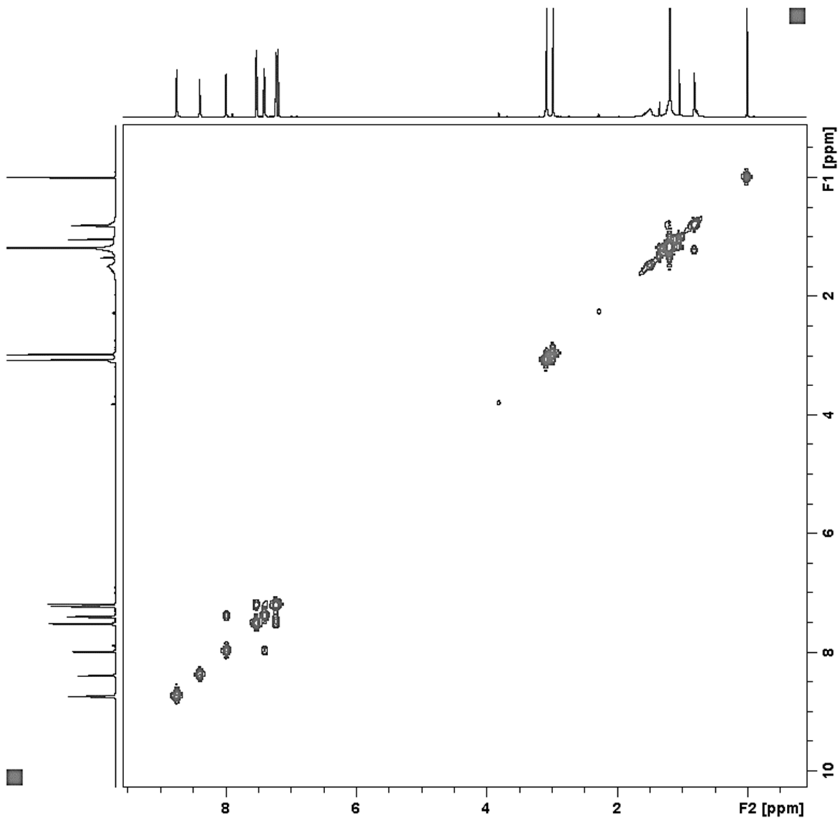

Figure S7. ^{1}H-^{1}H COSY of compound 8

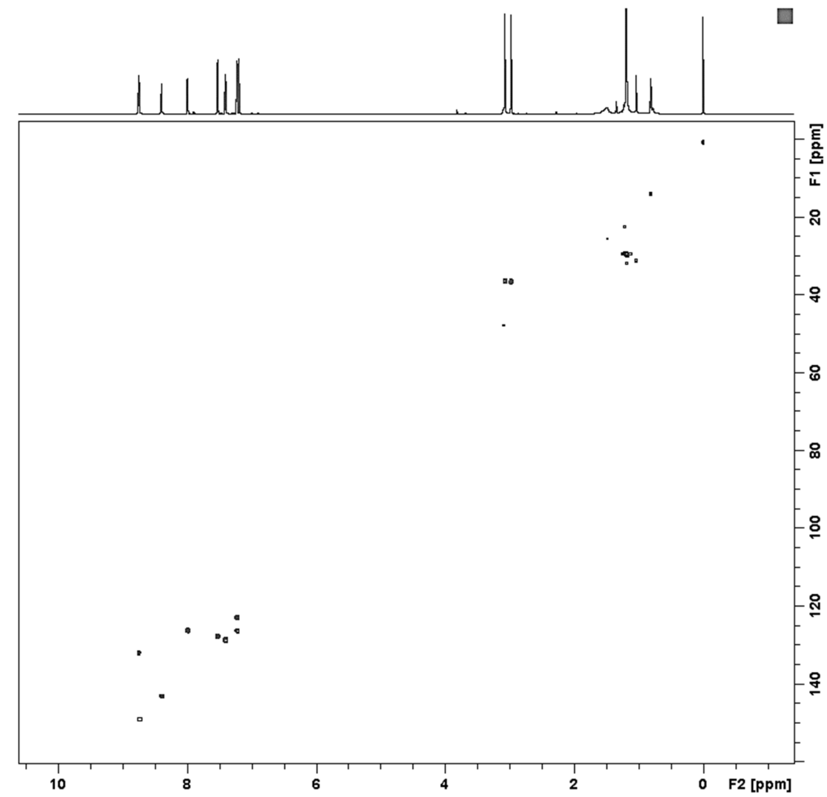

Figure S8. (^{1}H,^{13}C)-HSQC of compound 8

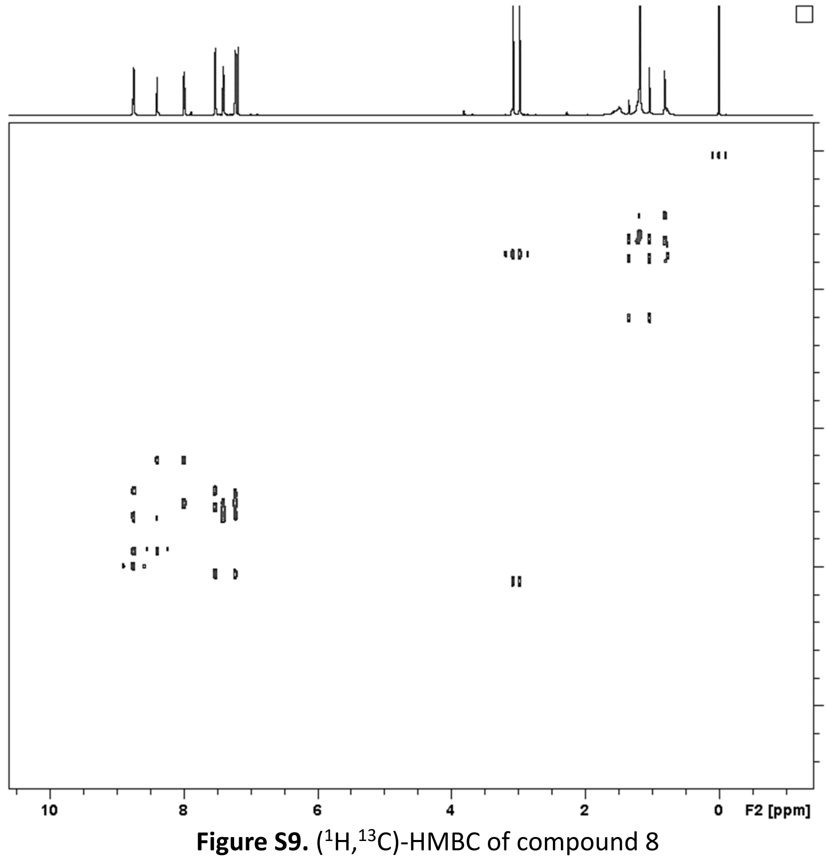

Figure S9. (^{1}H,^{13}C)-HMBC of compound 8

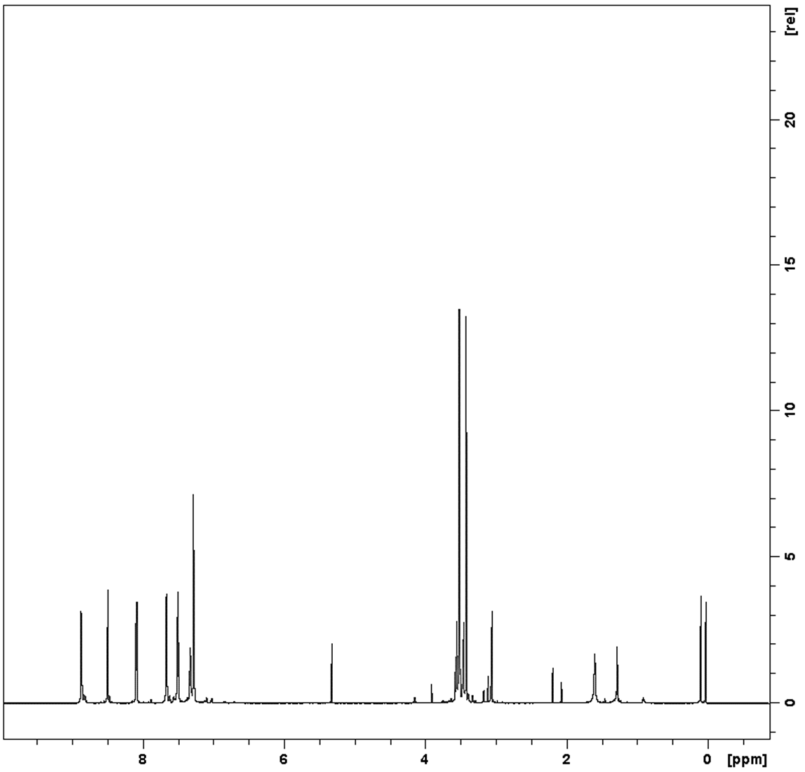

Figure S10. ^{1}H-NMR of compound 9

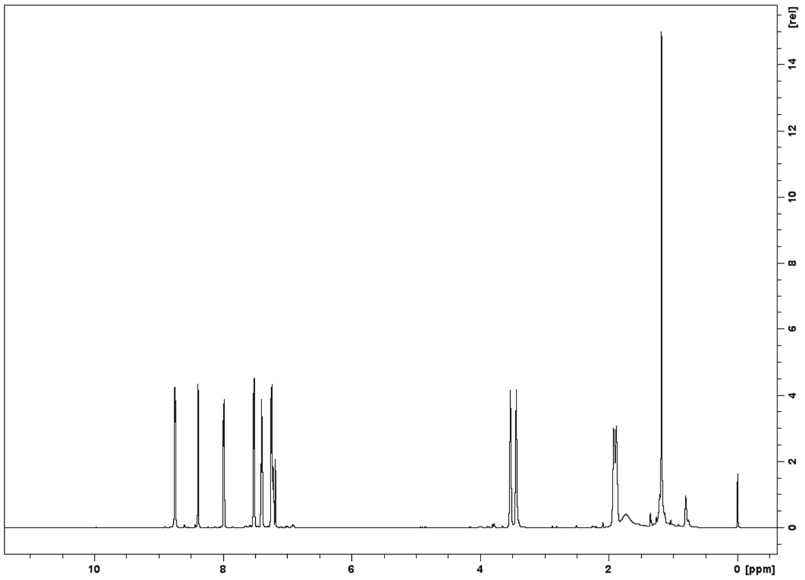

Figure S11. ^{1}H-NMR of compound 10

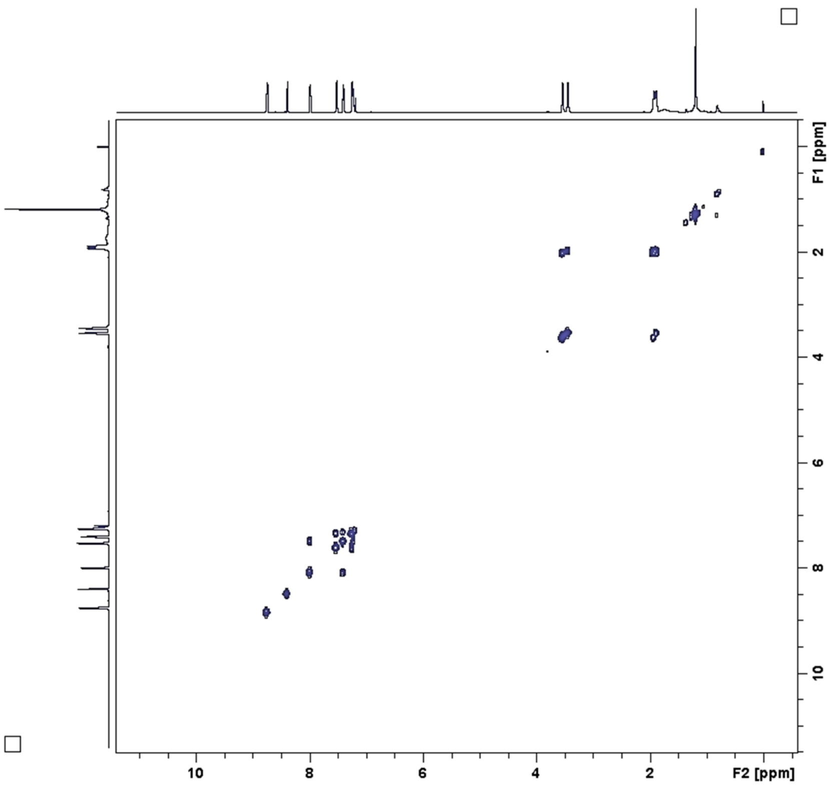

Figure S12. ^{1}H-^{1}H COSY of compound 10

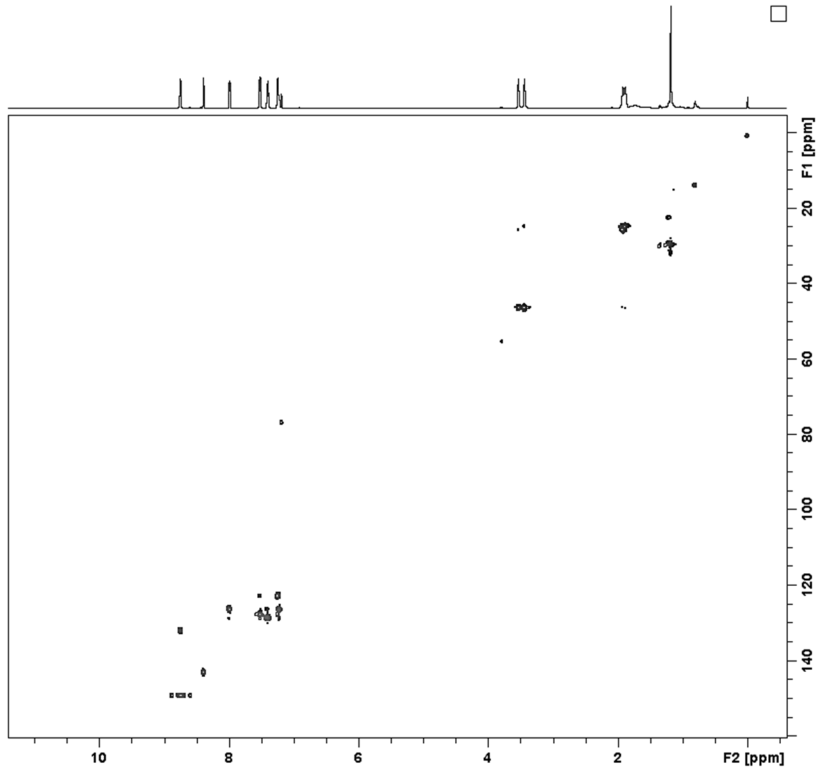

Figure S13. (^{1}H,^{13}C)-HSQC of compound 10

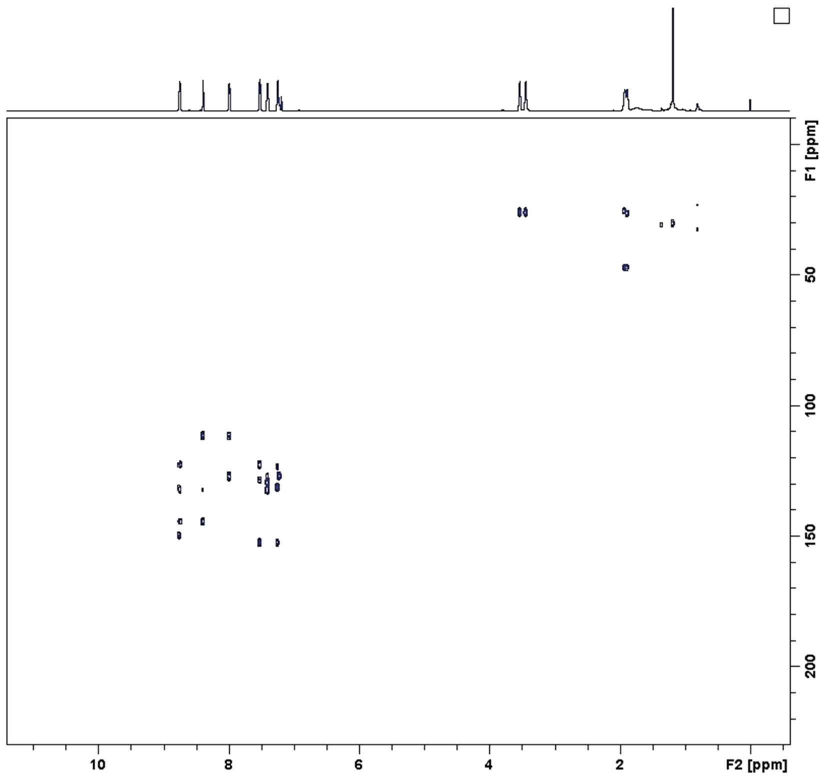

Figure S14. (^{1}H,^{13}C)-HMBC of compound 10

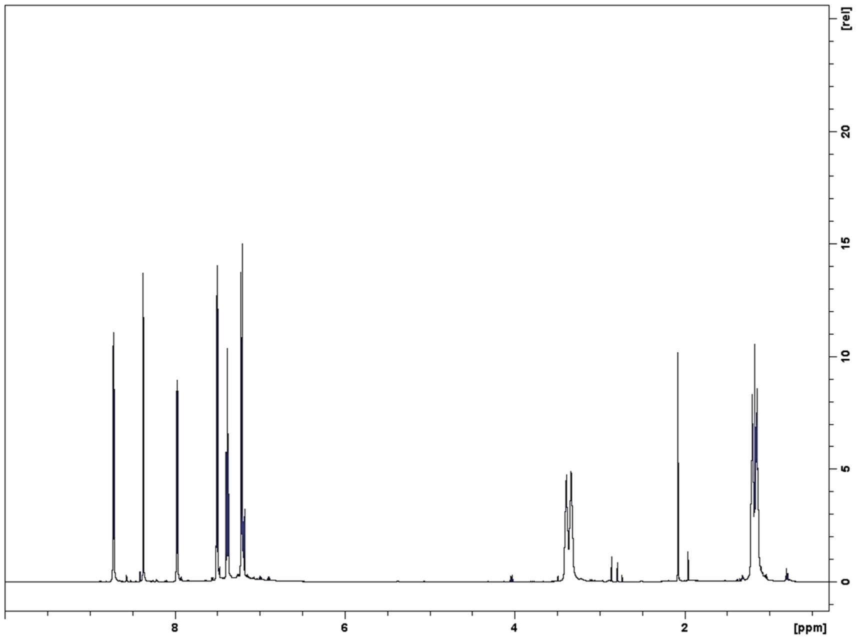

Figure S15. ^{1}H-NMR of compound 11

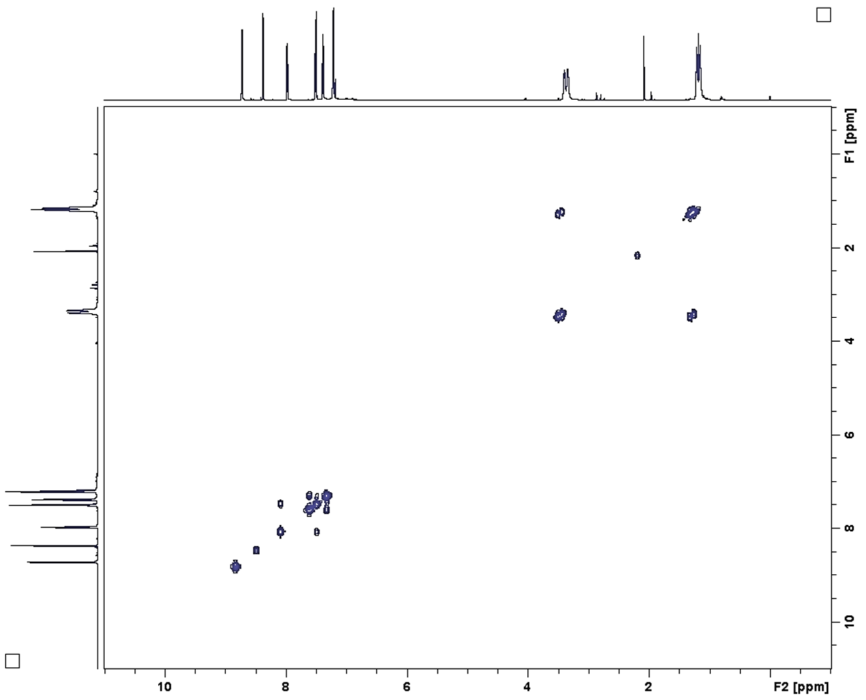

Figure S16. ^{1}H-^{1}H COSY of compound 11

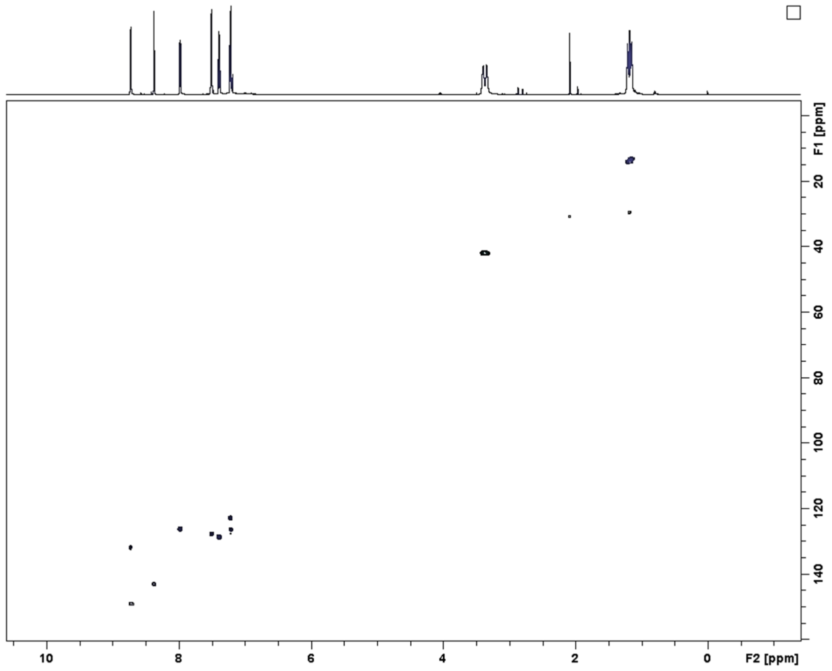

Figure S17. (^{1}H,^{13}C)-HSQC of compound 11

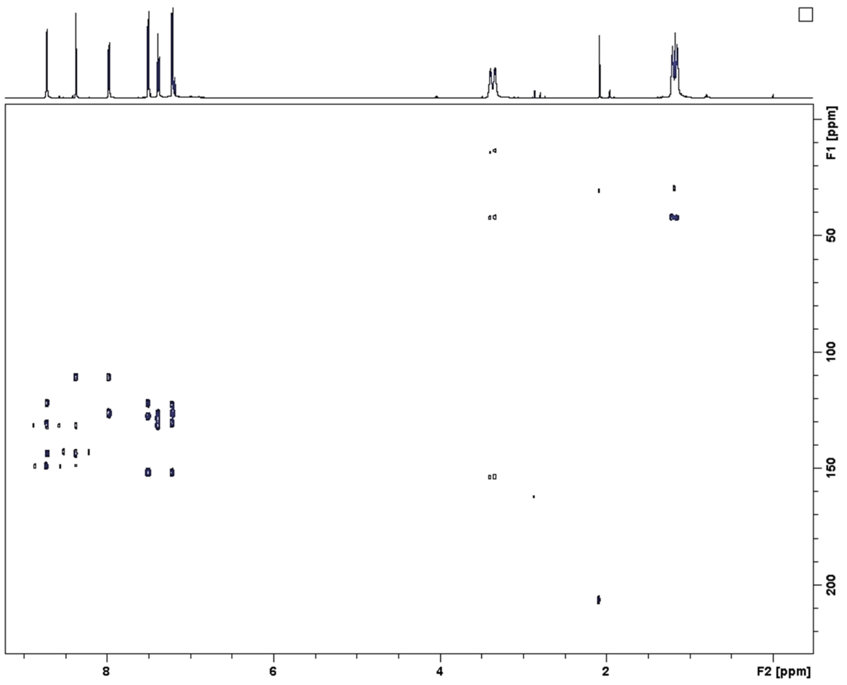

Figure S18. (1H,13C)-HMBC of compound 11

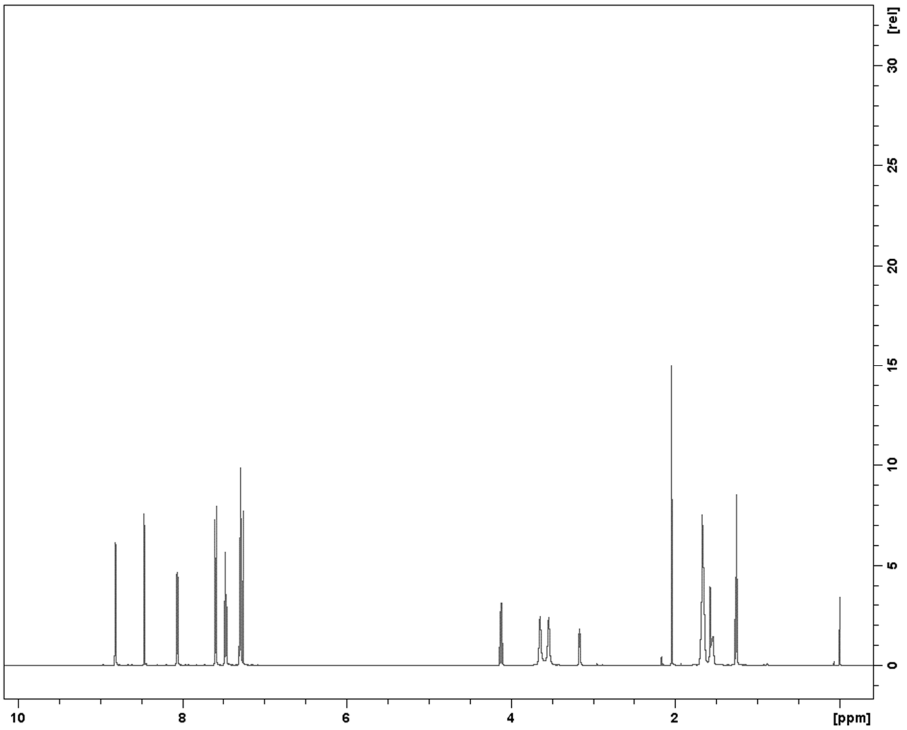

Figure S19. ^{1}H-NMR of compound 12

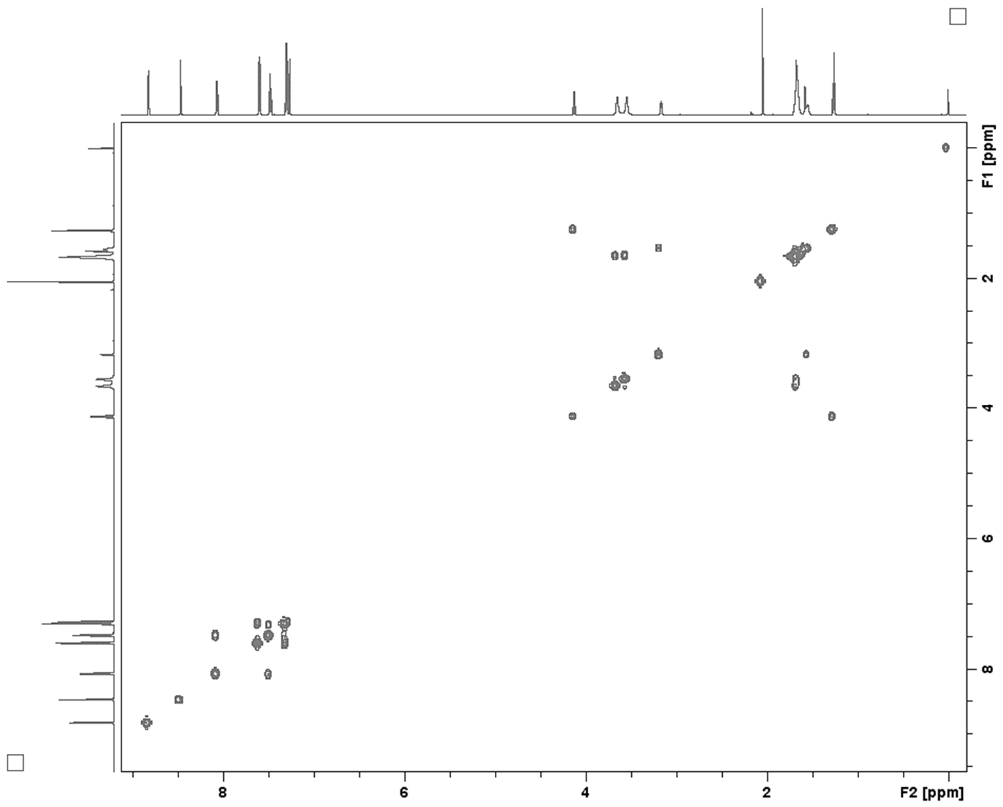

Figure S20. ^{1}H-^{1}H COSY of compound 12

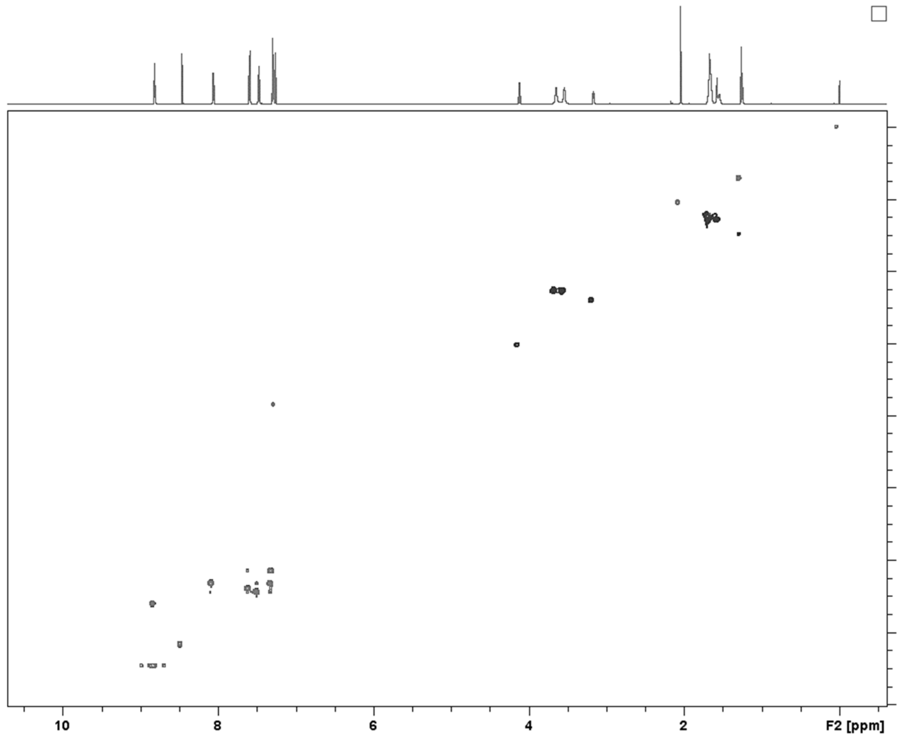

Figure S21. **(**^{1}H,$^{\mathbf{13}}C$)-HSQC of compound 12

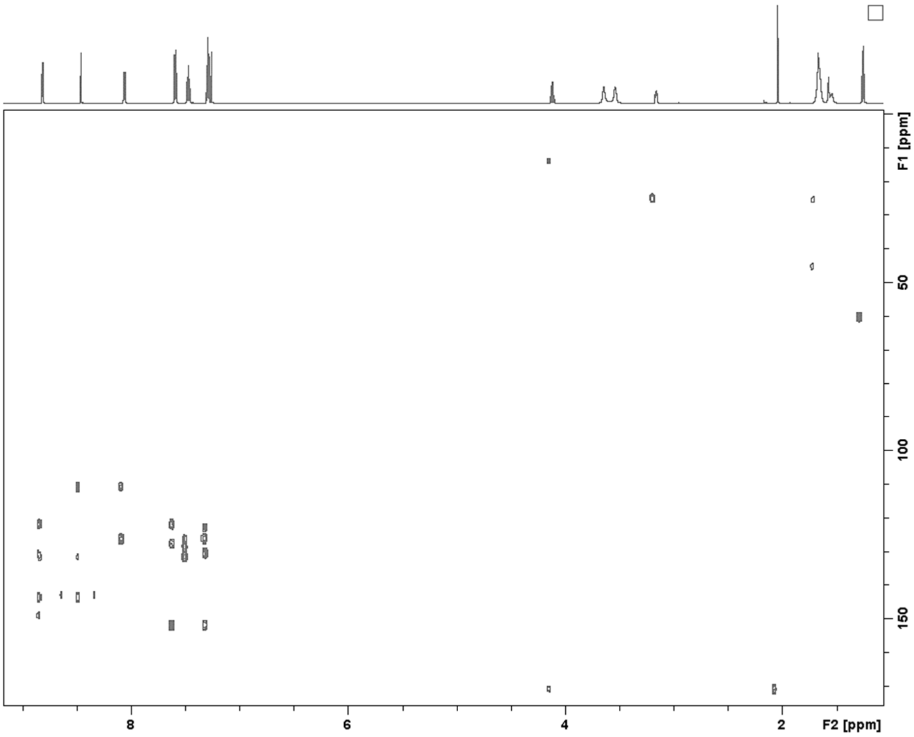

Figure S22. (^{1}H,^{13}C)-HMBC of compound 12

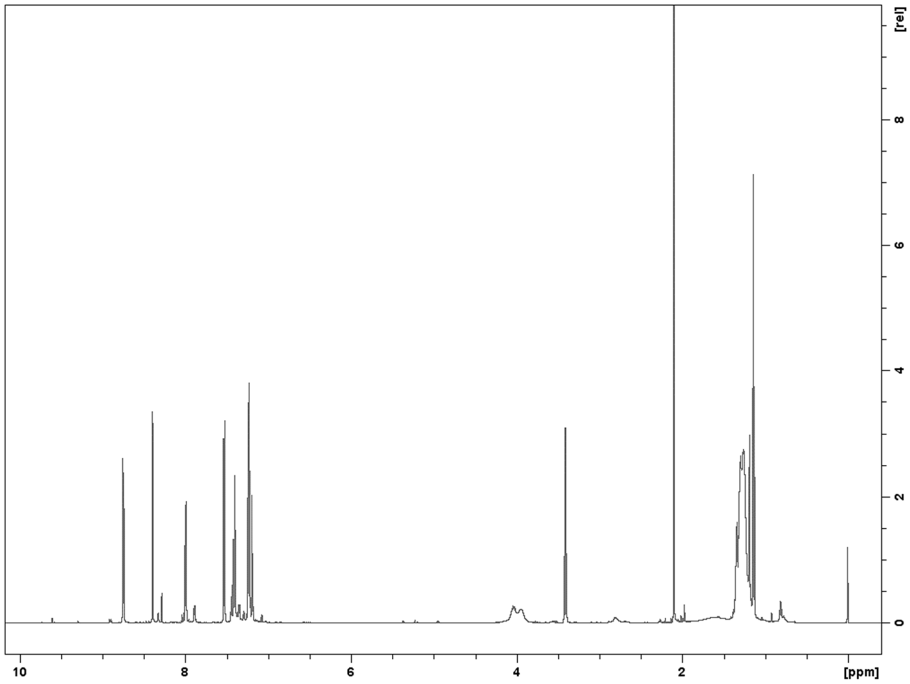

Figure S23. 1H-NMR of compound 13

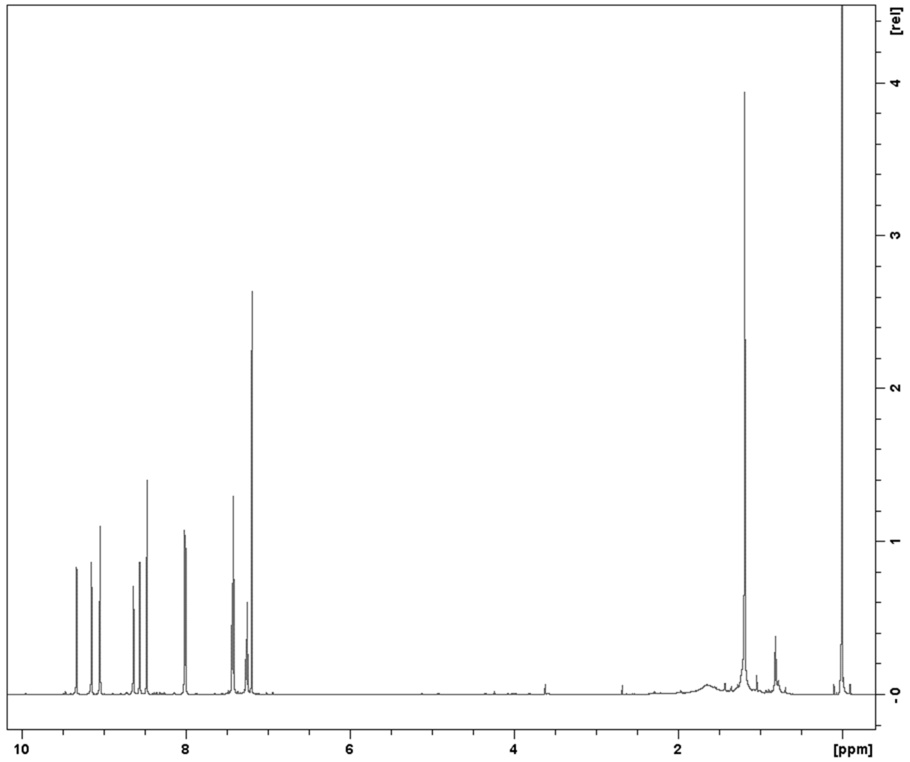

Figure S24. 1H-NMR of compound 14

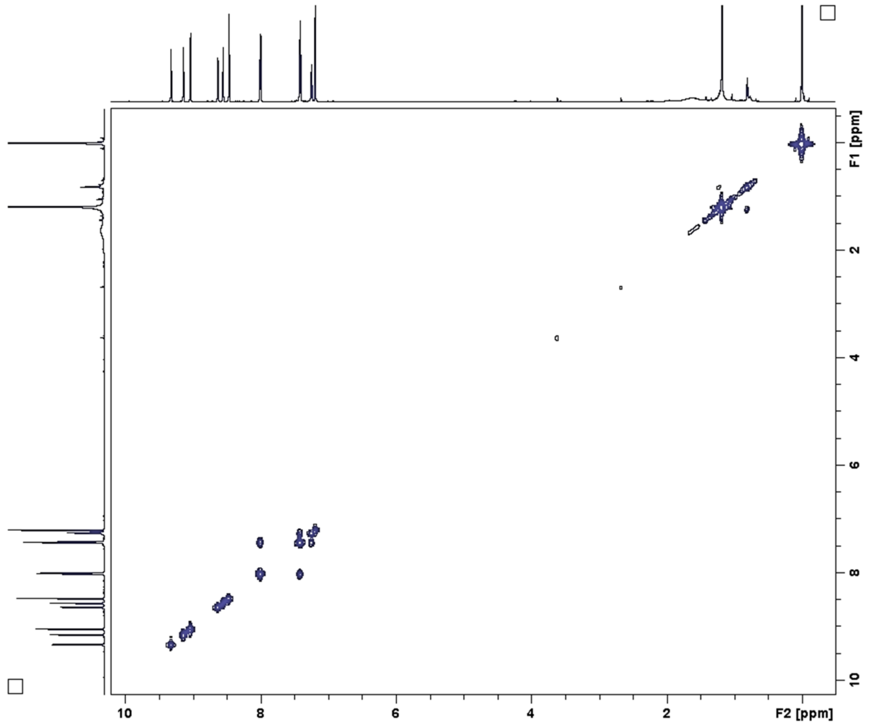

Figure S25. ^{1}H-^{1}H COSY of compound 14

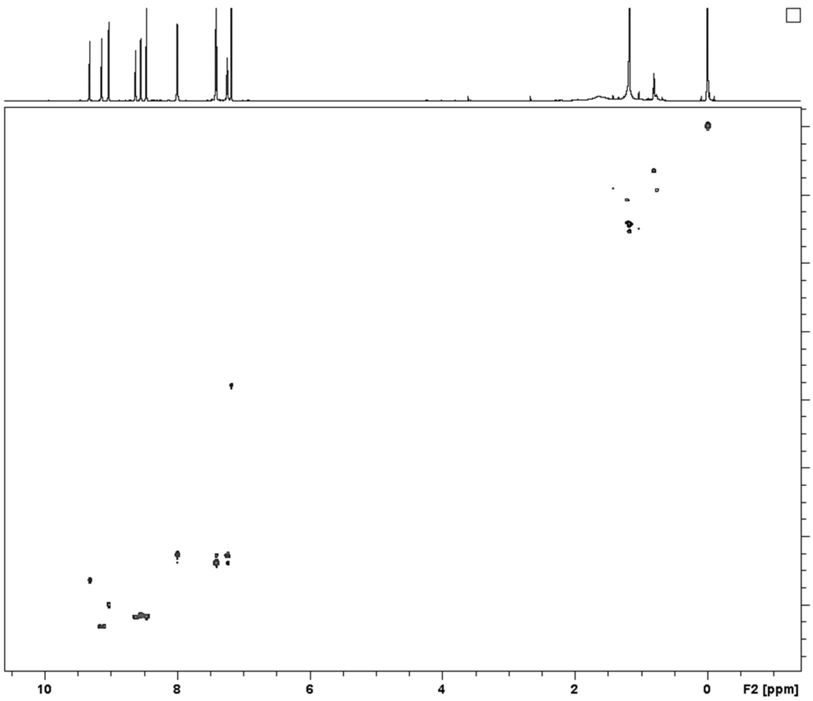

Figure S26. (^{1}H,**^{13}**C)-HSQC of compound 14

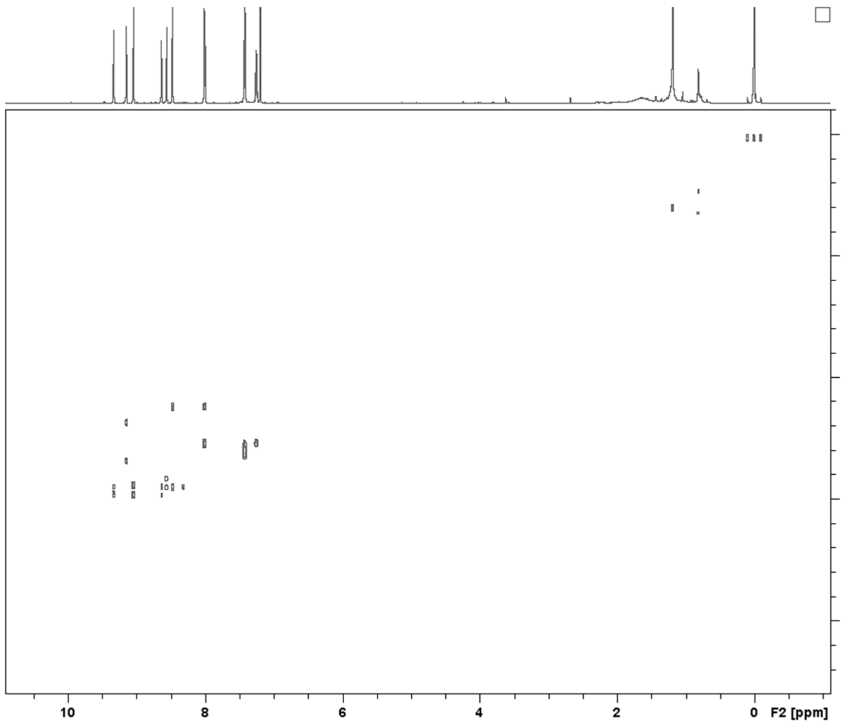

Figure S27. (^{1}H,^{13}C)-HMBC of compound 14

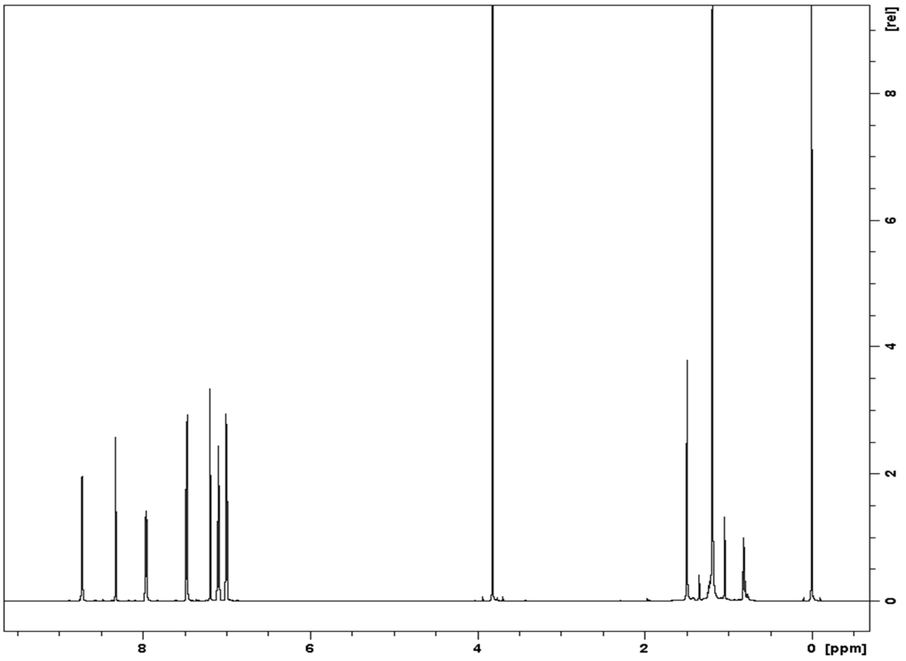

Figure S28. ^{1}H-NMR of compound 17

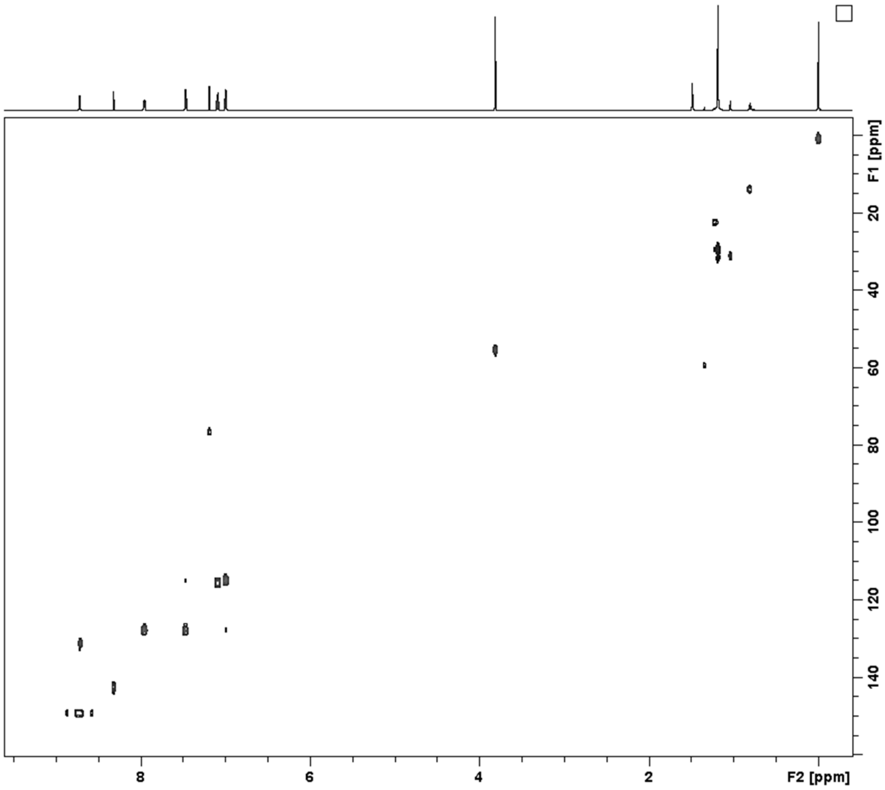

Figure S29. (^{1}H,^{13}C)-HSQC of compound 17

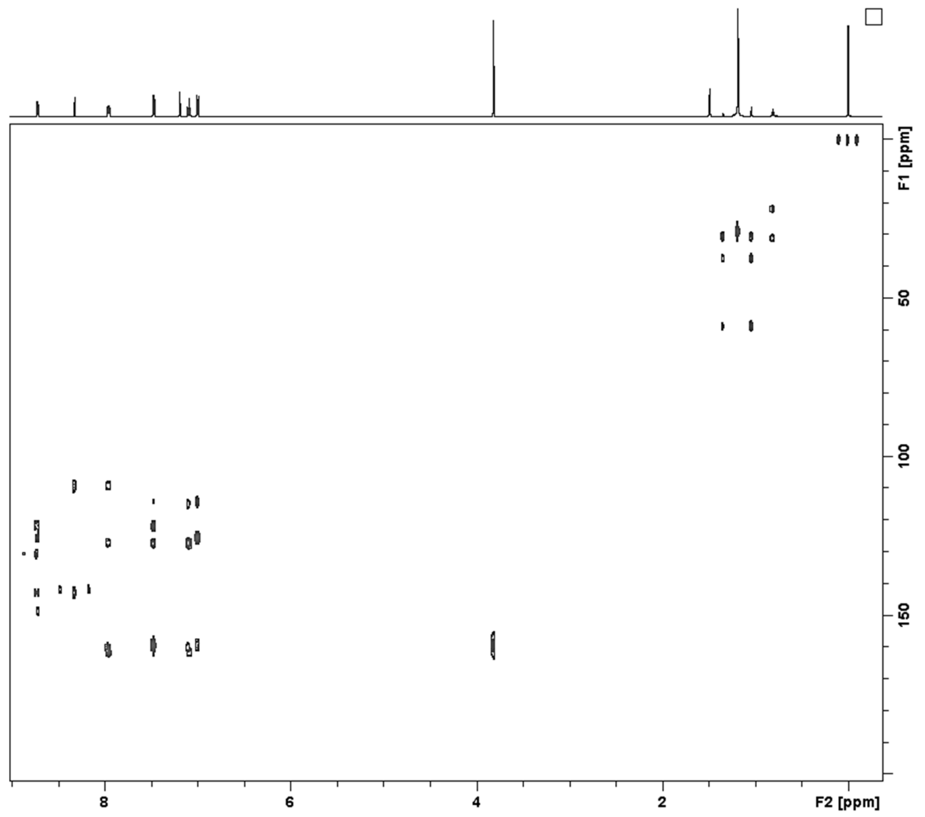

Figure S30. (^{1}H,^{13}C)-HMBC of compound 17

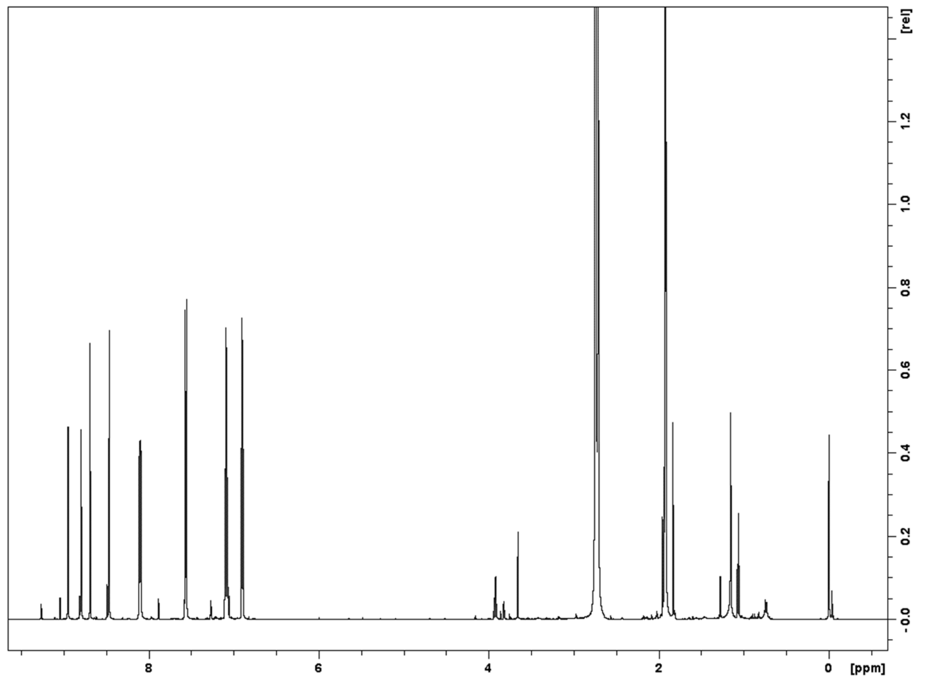

Figure S31. ^{1}H-NMR of compound 18

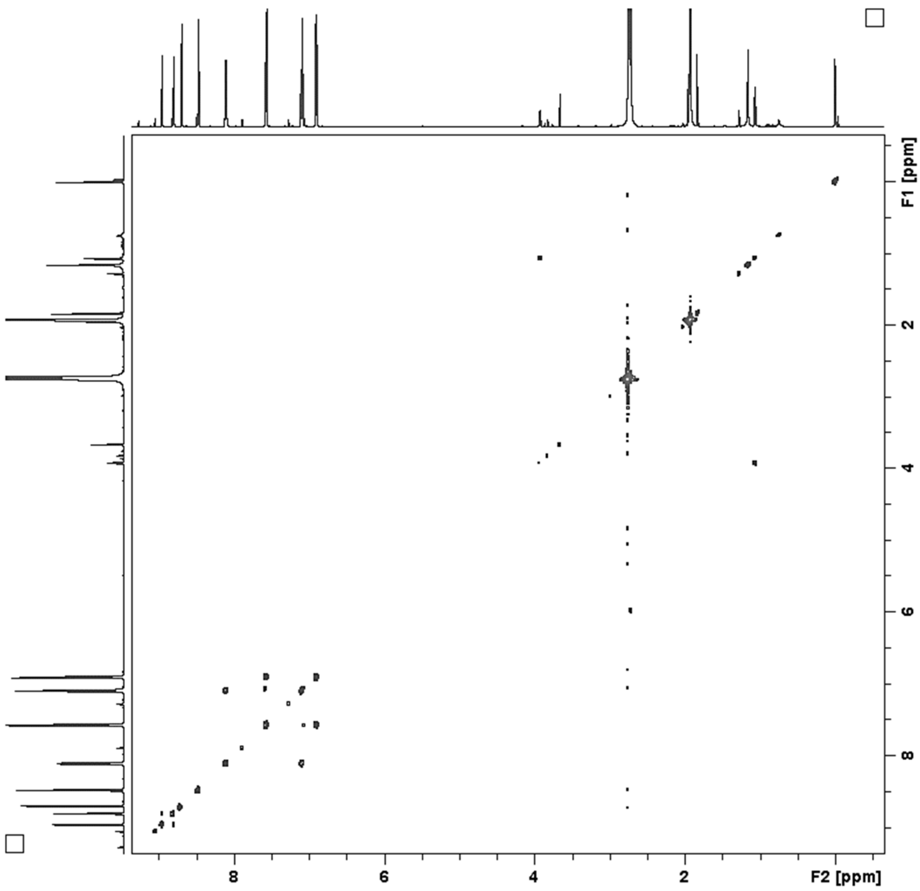

Figure S32. ^{1}H-^{1}H COSY of compound 18

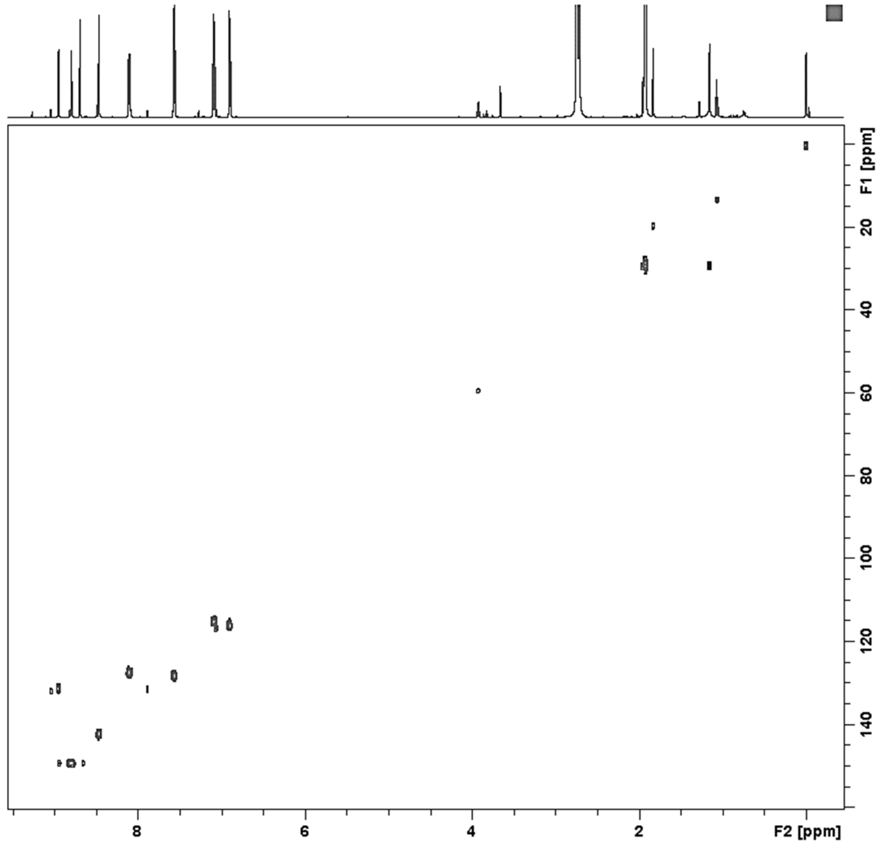

Figure S33. **(**^{1}H,$^{\mathbf{13}}C$)-HSQC of compound 18

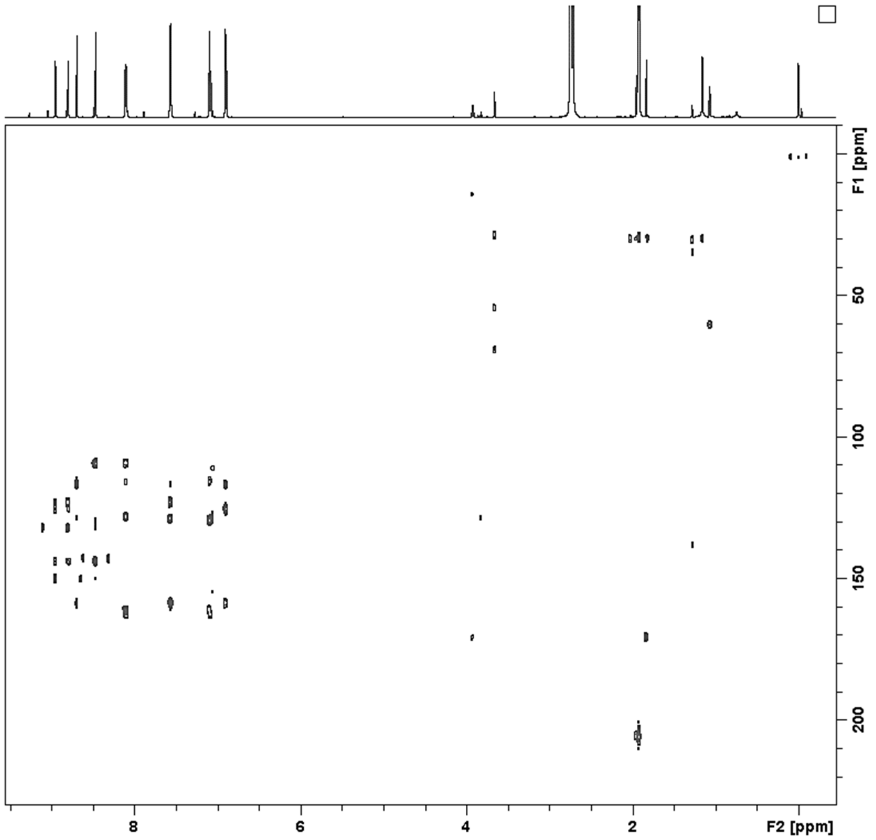

Figure S34. (^{1}H,^{13}C)-HMBC of compound 18

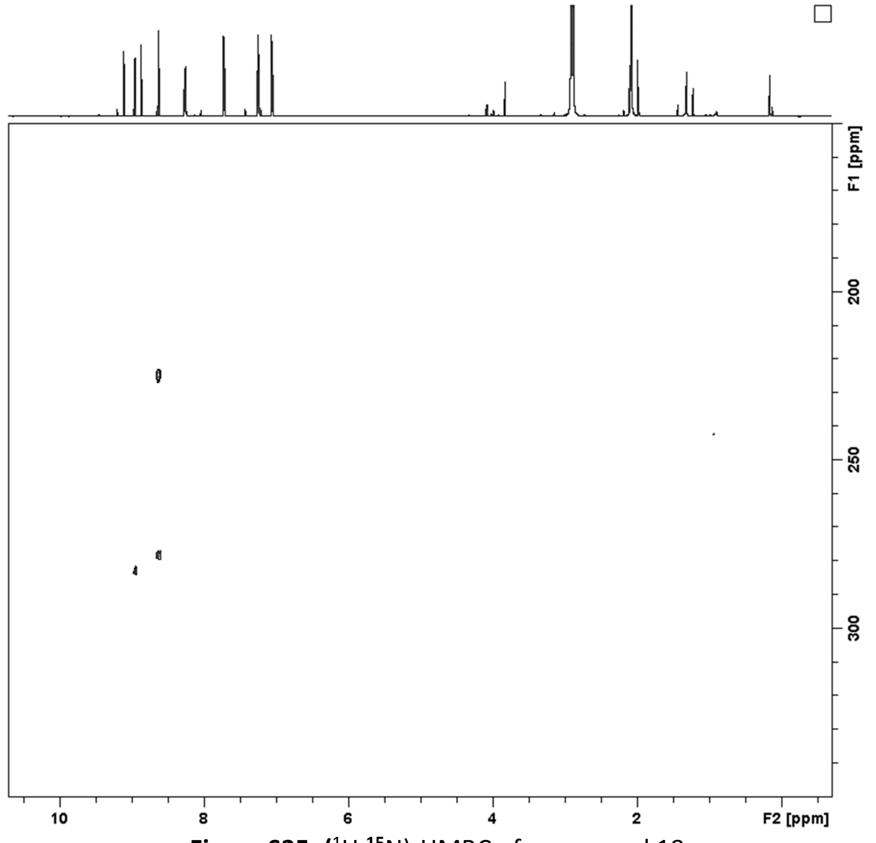

Figure S35. (^{1}H,^{15}N)-HMBC of compound 18

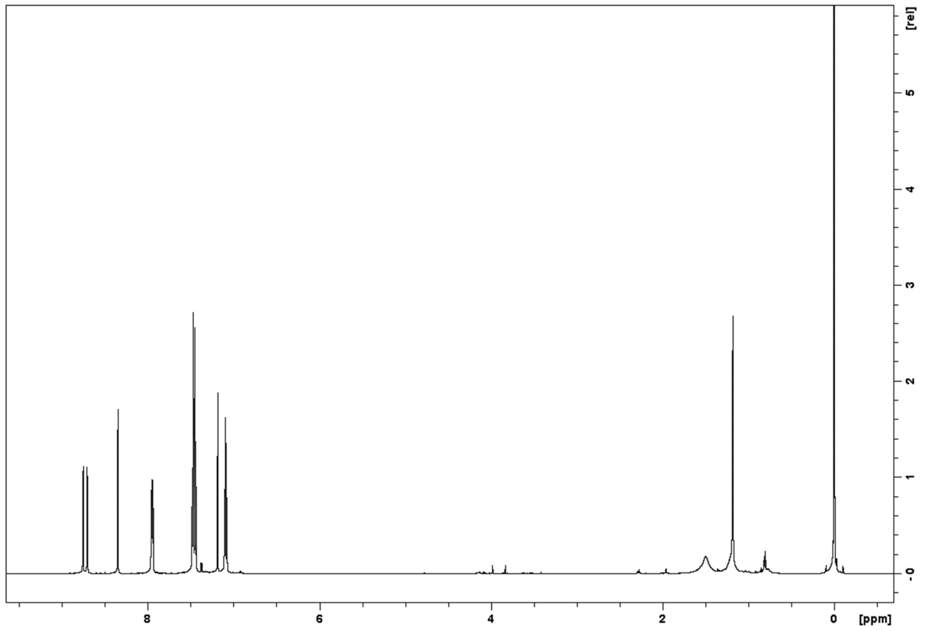

Figure S36. ^{1}H-NMR of compound 19

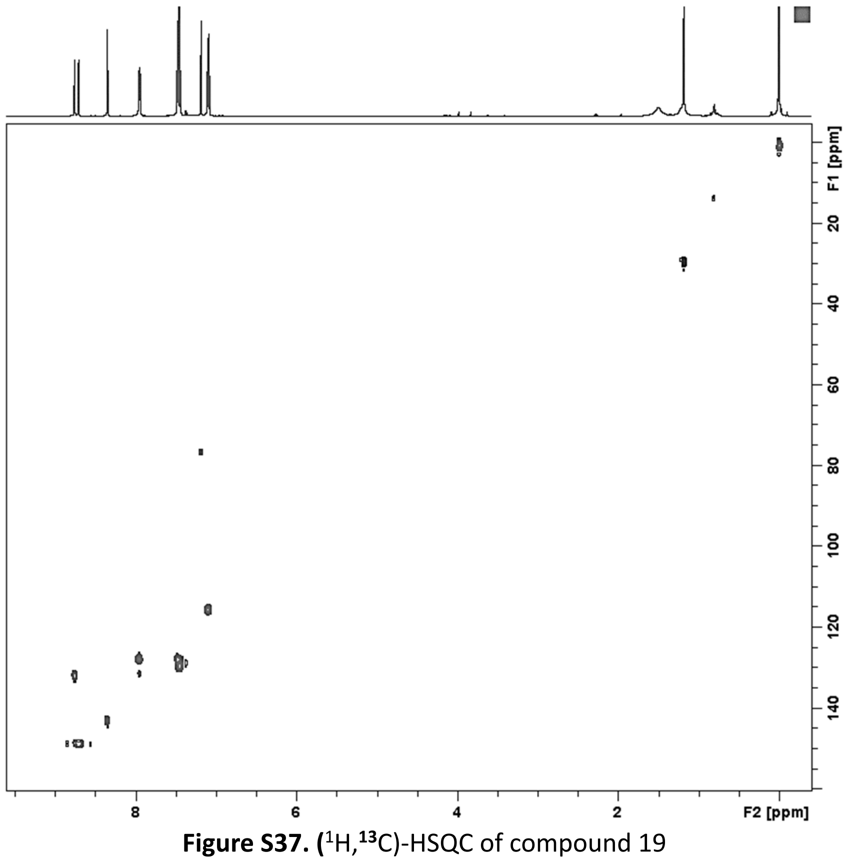

Figure S37. (^{1}H,^{13}C)-HSQC of compound 19

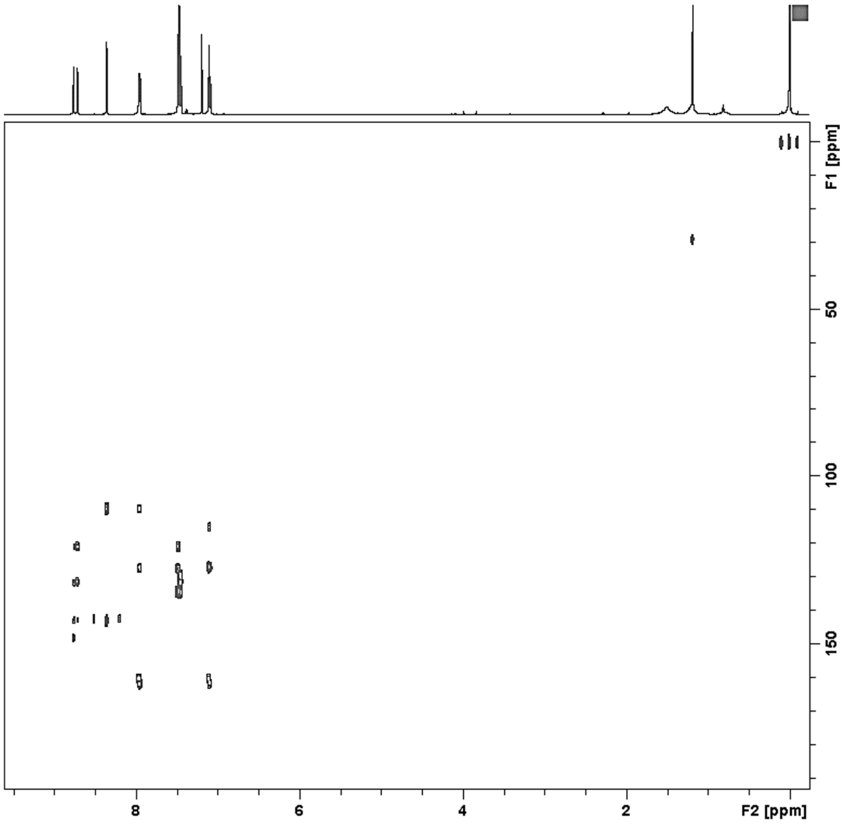

Figure S38. (^{1}H,^{13}C)-HMBC of compound 19

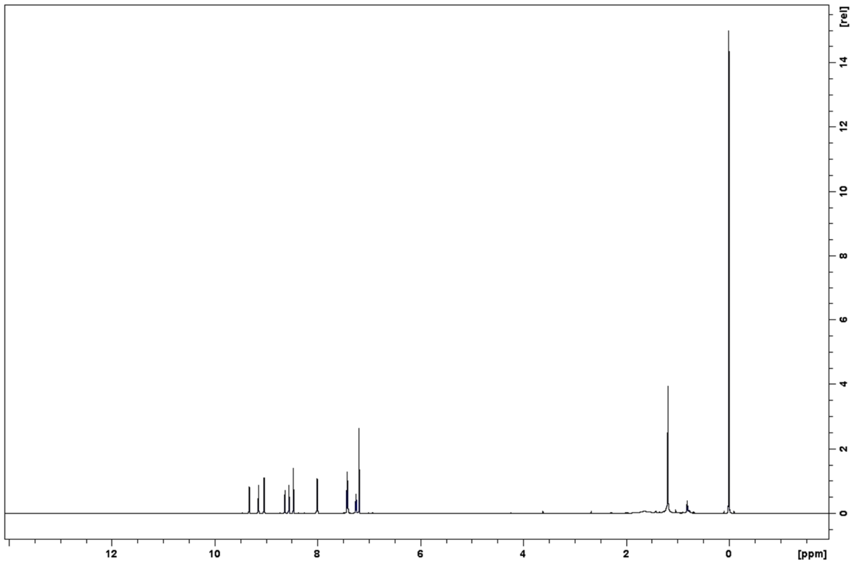

Figure S39. ^{1}H-NMR of compound 21

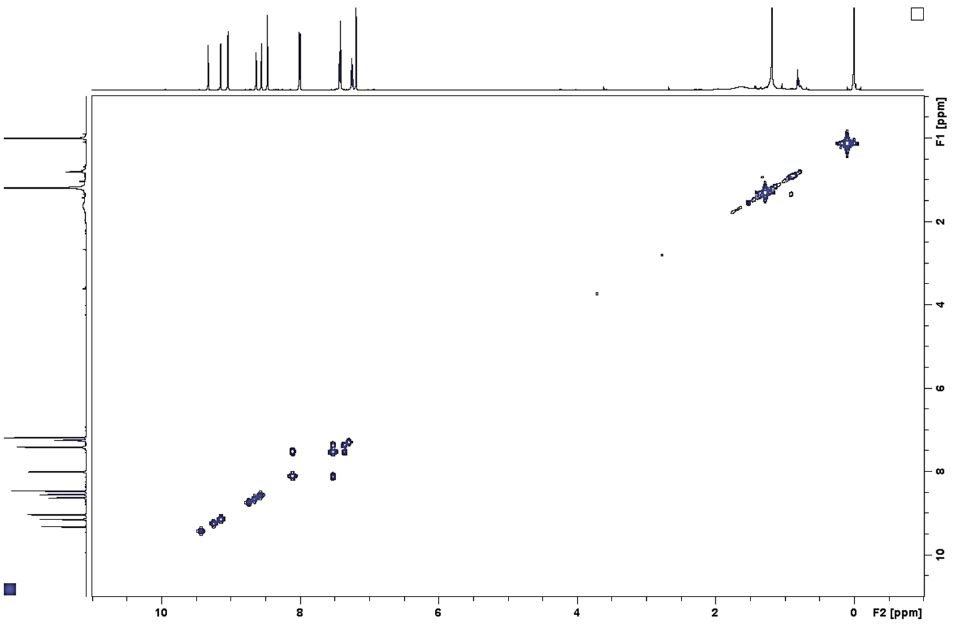

Figure S40. ^{1}H-^{1}H COSY of compound 21

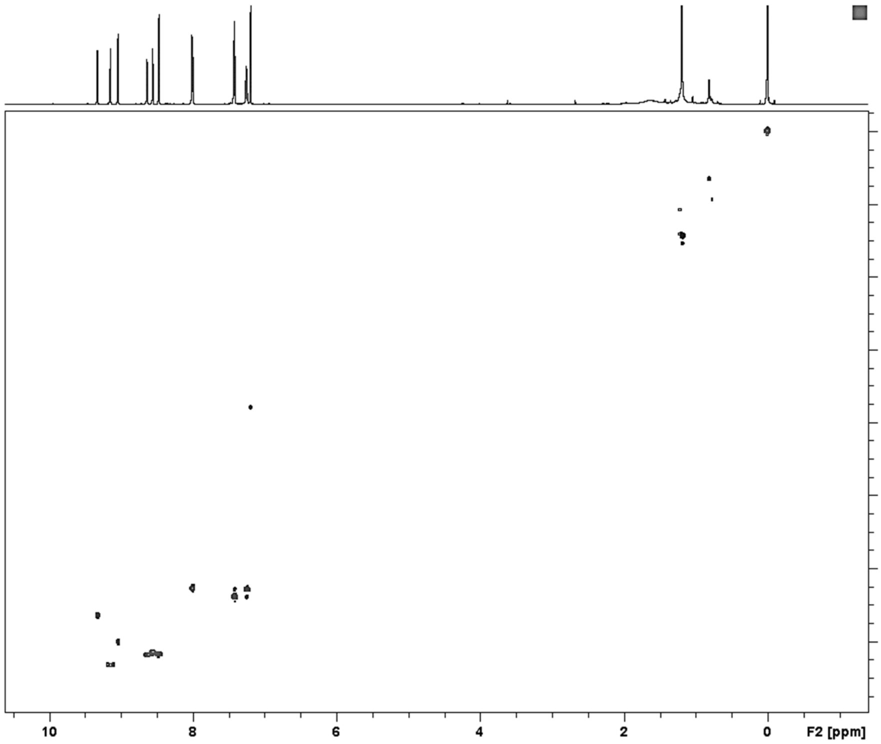

Figure S41. (^{1}H,^{13}C)-HSQC of compound 21

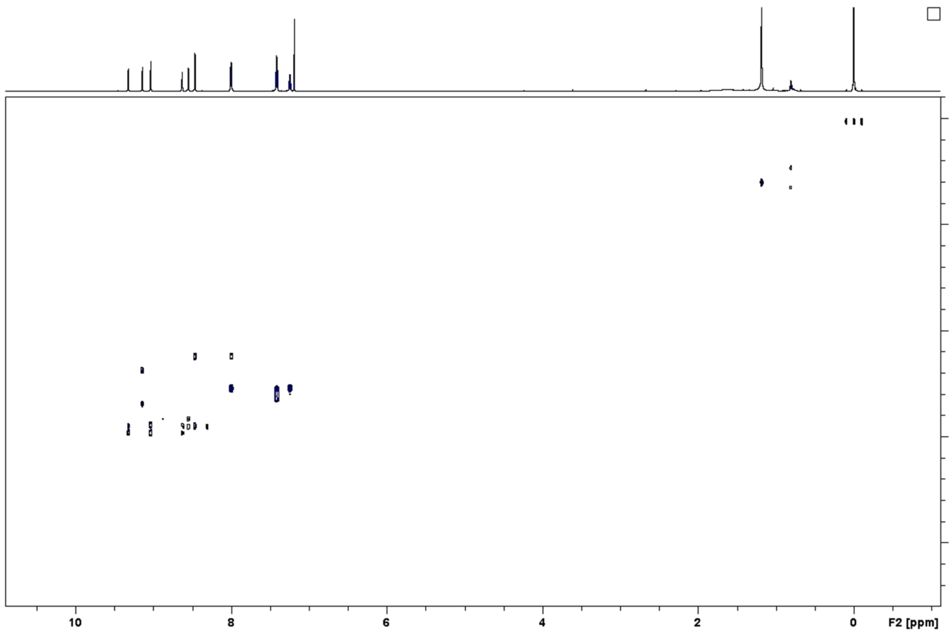

Figure S42. (^{1}H,^{13}C)-HMBC of compound 21

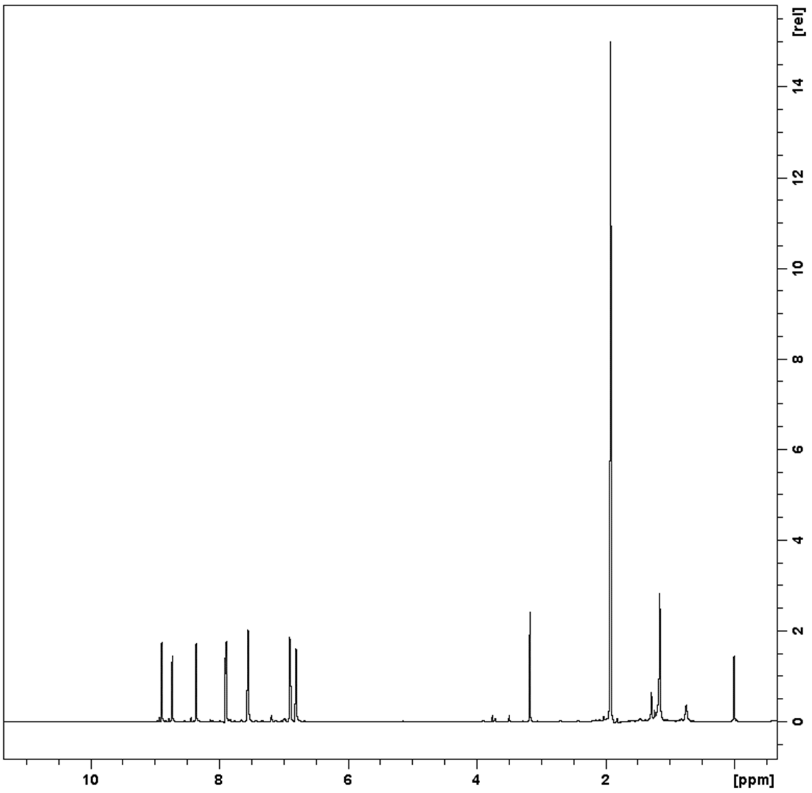

Figure S43. ^{1}H-NMR of compound 22

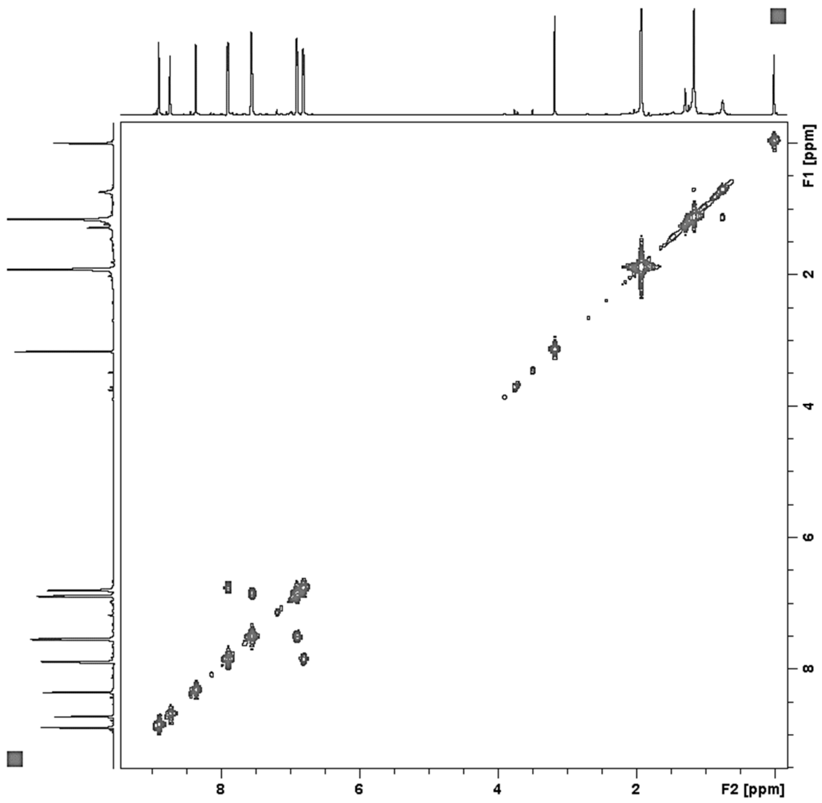

Figure S44. ^{1}H-^{1}H COSY of compound 22

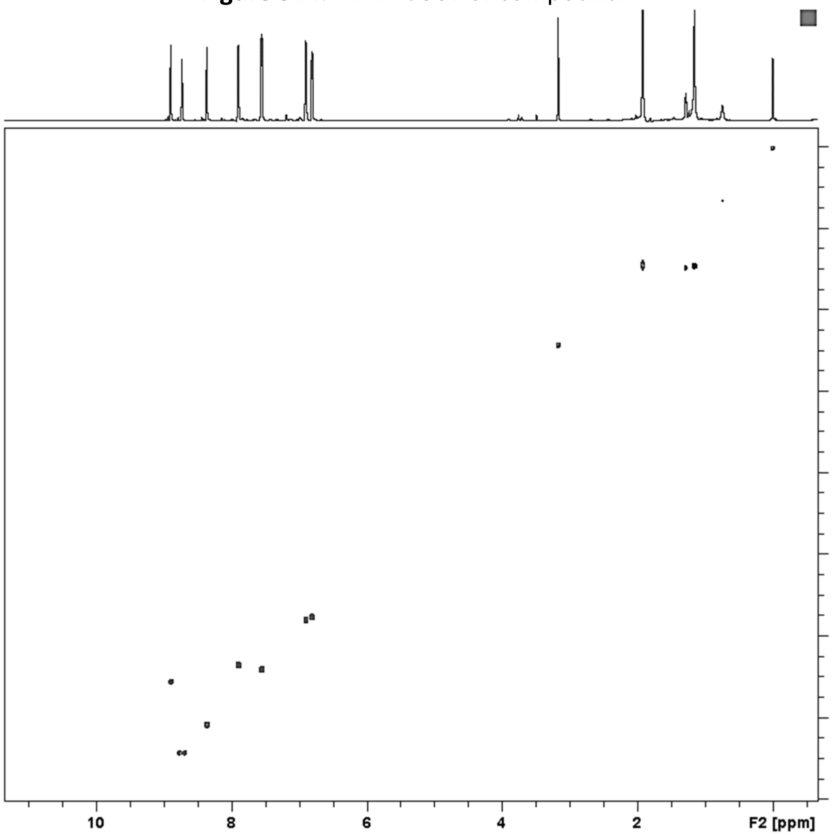

Figure S45. (^{1}H,^{13}C)-HSQC of compound 22

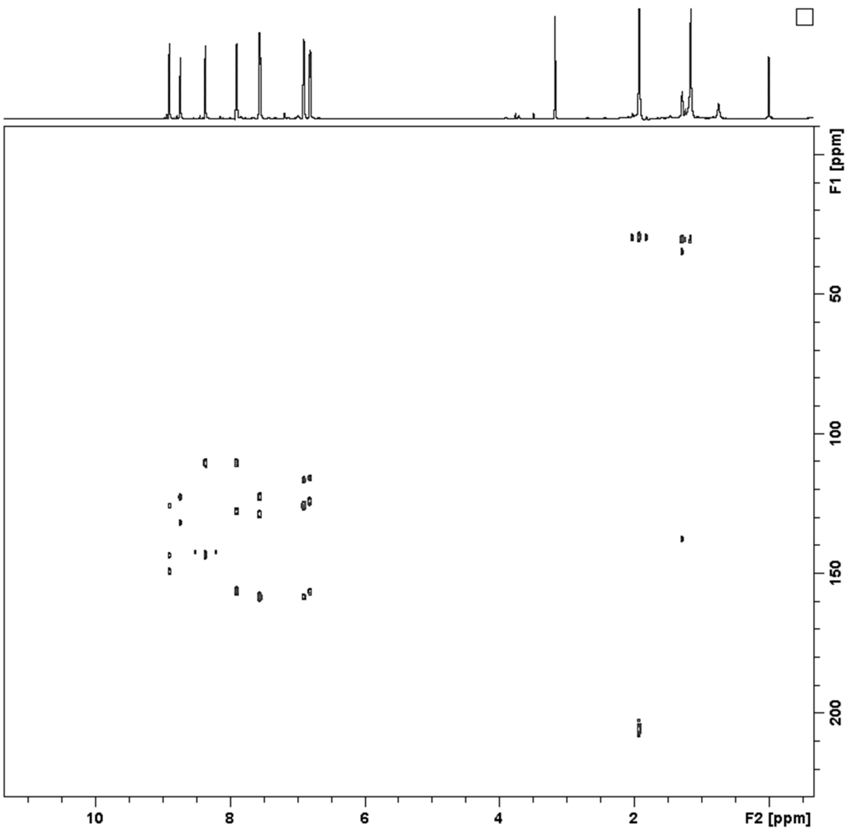

Figure S46. (^{1}H,^{13}C)-HMBC of compound 22

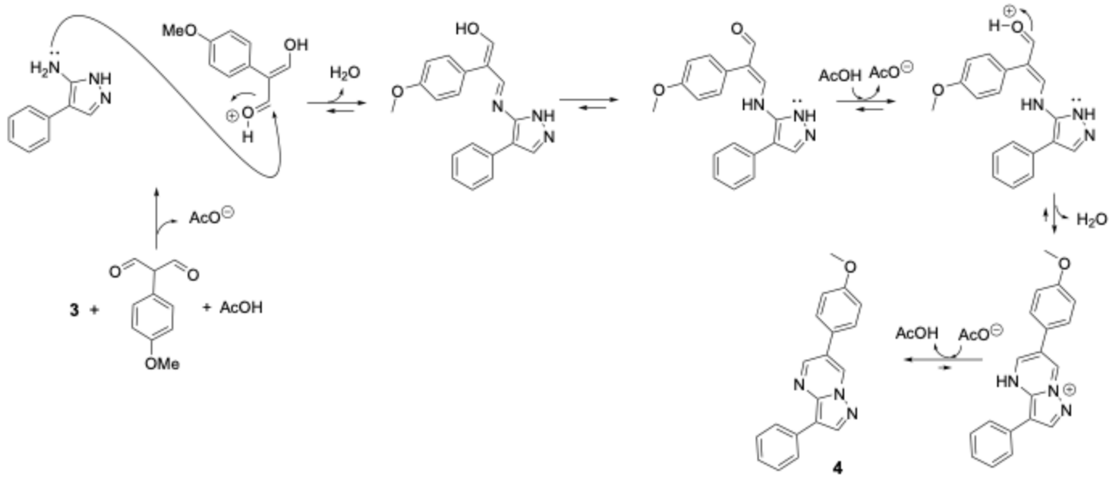

Figure S47. Mechanism of formation of pyrazolopyrimidine **4**

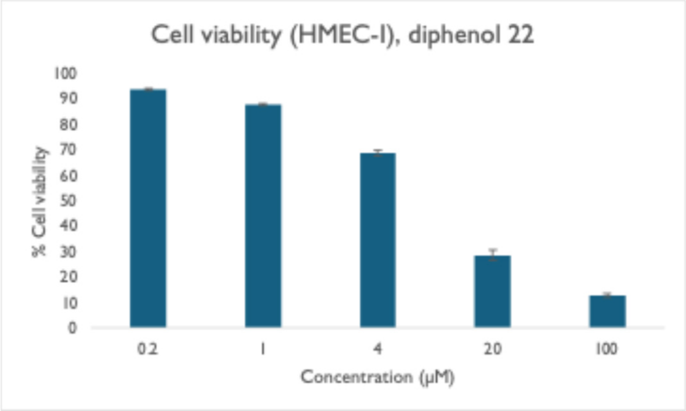

Figure S48: Cell viability (%) diagram for diphenol 22 for HMEC-1 cells

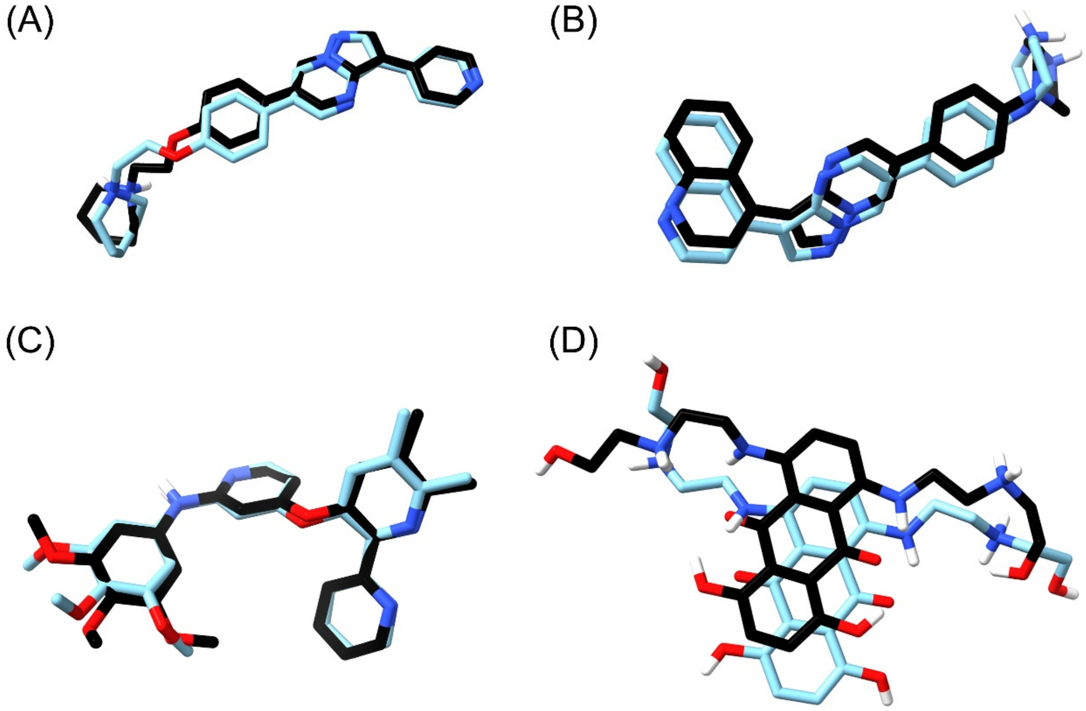

Figure S49: Calculated conformations of the reference ligands in the different protein targets. (A) compound TAK in complex with the AMPK receptor, (B) compound ZZG in complex with the ACVR1 kinase domain, and (C) compound 15 in complex with the TGFβ type I receptor and (D) compound MIX in complex with the ABCG2 transporter. Crystallographic conformations are in black color. RMSDs between the predicted and crystallographic conformations were calculated by means of the LigRMSD server [1] and were found equal to 1.18 Å, 0.90 Å, 0.57 Å and 2.19 Å, respectively.

References

1. Velázquez-Libera, J.L.; Durán-Verdugo, F.; Valdés-Jiménez, A.; Núñez-Vivanco, G.; Caballero, J. LigRMSD: A web server for automatic structure matching and RMSD calculations among identical and similar compounds in protein-ligand docking. *Bioinformatics* **2020**, *36*, 2912–2914. https://doi.org/10.1093/bioinformatics/btaa018.
